# De-biasing microbiome sequencing data: bacterial morphology-based correction of extraction bias and correlates of chimera formation

**DOI:** 10.1186/s40168-024-01998-4

**Published:** 2025-02-04

**Authors:** Luise Rauer, Amedeo De Tomassi, Christian L. Müller, Claudia Hülpüsch, Claudia Traidl-Hoffmann, Matthias Reiger, Avidan U. Neumann

**Affiliations:** 1 Institute of Environmental Medicine and Integrative Health, Faculty of Medicine, University of Augsburg, Augsburg, Germany; 2 Chair of Environmental Medicine, Technical University of Munich, Munich, Germany; 3 Institute of Environmental Medicine, Helmholtz Munich, Augsburg, Germany; 4 Institute of Computational Biology, Helmholtz Munich, Neuherberg, Germany; 5 Department of Statistics, Ludwig Maximilian University of Munich, Munich, Germany; 6 Center for Computational Mathematics, Flatiron Institute, New York, USA; 7 CK CARE, Christine Kühne Center for Allergy Research and Education, Davos, Switzerland; 8 ZIEL - Institute for Food & Health, Technical University of Munich, Freising-Weihenstephan, Germany

**Keywords:** Bacterial mock community, Positive control, Extraction bias, Cell lysis, Kitome, Contamination, Chimera formation, Bias correction, 16S rRNA gene sequencing

## Abstract

**Introduction:**

Microbiome amplicon sequencing data are distorted by multiple protocol-dependent biases from bacterial DNA extraction, contamination, sequence errors, and chimeras, hindering clinical microbiome applications. In particular, extraction bias is a major confounder in sequencing-based microbiome analyses, with no correction method available to date. Here, we suggest using mock community controls to computationally correct extraction bias based on bacterial morphological properties.

**Methods:**

We compared dilution series of 3 cell mock communities with an even or staggered composition. DNA of these mock, and additional skin microbiome samples, was extracted with 8 different extraction protocols (2 buffers, 2 extraction kits, 2 lysis conditions). Extracted DNA was sequenced (V1–V3 16S rRNA gene) together with corresponding DNA mocks.

**Results:**

Microbiome composition was significantly different between extraction kits and lysis conditions, but not between buffers. Independent of the extraction protocol, chimera formation increased with higher input cell numbers. Contaminants originated mostly from buffers, and considerable cross-contamination was observed in low-input samples. Comparing the microbiome composition of the cell mocks to corresponding DNA mocks revealed taxon-specific protocol-dependent extraction bias. Strikingly, this extraction bias per species was predictable by bacterial cell morphology. Morphology-based computational correction of extraction bias significantly improved resulting microbial compositions when applied to different mock samples, even with different taxa. Equivalent correction of the skin samples showed a substantial impact on microbiome compositions.

**Conclusions:**

Our results indicate that higher DNA density increases chimera formation during PCR amplification. Furthermore, we show that computational correction of extraction bias based on bacterial cell morphology would be feasible using appropriate positive controls, thus constituting an important step toward overcoming protocol biases in microbiome analysis.

Video Abstract

**Supplementary Information:**

The online version contains supplementary material available at 10.1186/s40168-024-01998-4.

## Introduction

Investigating the human microbiome with 16S rRNA targeted gene sequencing has become integral to environmental and medical scientific studies. However, the lack of reproducibility and generalizability of results is increasingly recognized as one of the key challenges in microbiome research. This inconsistency in findings can be attributed to the wide variety of methods available for generating sequencing-based microbiome data. This variability in methods and the biases associated with each step and method choice, from sampling to sequencing, critically limit the comparability of microbiome studies [1] and finally hamper finding robust clinical microbiome applications [2].

The most impactful biases originate from DNA extraction, contamination, amplification and sequencing errors, and chimeras. Amplification and sequencing artifacts such as sequence errors and chimeras can critically impact taxonomic annotation and inflate diversity estimates. Chimera formation remains an inherent problem in multi-template PCR reactions with high homology between templates, as in 16S rRNA gene sequencing experiments [3]. Computational pipelines can be used to partially correct sequence errors (e.g., DADA2 [4], deblur [5]) and remove chimeras (e.g., UCHIME [6], ChimeraSlayer [7]) with mixed success [8, 9]. Similarly, contaminants frequently originate from lab reagents and operators during DNA extraction and PCR amplification [10]. Besides external DNA contamination, internal cross-contamination by index hopping and bleed-through may significantly blur microbial signatures but is rarely investigated [11]. Despite laboratory efforts and new computational approaches [12, 13], both contamination and cross-contamination remain particularly problematic for low biomass samples [10, 11, 14, 15], such as from milk, lung, or skin microbiomes [16].

However, the data is probably most confounded by bias introduced during DNA extraction, covering the process of cell lysis and isolation of bacterial DNA. Several protocols have been tested and optimized for specific sample types and low-biomass environments, combining different extraction kits, preservative buffers, and cell lysis approaches [17–20]. Accordingly, existing extraction approaches differ in their cell lysis efficiency, DNA yield, DNA purity, DNA integrity, reproducibility of results, and species richness [21, 22]. Most critically, however, extraction protocols differ in their ability to accurately reflect the original sample composition, and no protocol correctly recovers actual bacterial sample compositions due to differences in the lysis efficiency and DNA recovery of bacterial taxa [18, 20, 23–28]. Thus, extraction bias has a significant impact on the results of microbiome sequencing studies [17, 26, 29]. With striking differences in sample compositions between extraction protocols, extraction bias has been identified multiple times as one of the major confounders in microbiome sequencing studies [24, 30, 31]. Crucially, there is currently no laboratory or computational approach to handle extraction bias in environmental microbiome samples. Therefore, with no solution available to date, extraction bias remains one of the major problems in microbiome studies.

Each bias from extraction, amplification, sequencing, and contamination slightly distorts the data, accumulating over the entire microbiome data generation pipeline and multiplying into significant protocol-dependent biases [1]. The complete bias in microbiome sequencing experiments can be assessed with the help of standardized controls with known sample composition, like mock communities [32–34]. Using such mocks, the pioneering approach metacal [1, 35] provided a powerful computational model to assess and transfer bias between mock samples. However, it is crucial to note that metacal only allows for correcting bias between identical mock species. Therefore, it is currently limited by the size of positive controls, lacking generalizability to non-mock taxa present in environmental microbiomes. For metacal to be widely applicable and allow the transfer of bias from mock to environmental microbiome samples, the bias would need to be linked to taxon properties, which has not yet been done.

We designed this study to investigate the so-far unsolved extraction bias and its relation to the morphological properties of bacterial cells. In order to accurately quantify extraction bias, we also explore downstream protocol biases from (cross-)contamination, sequence errors, and chimera formation. Our analyses indicate that mock communities may be used to measure and computationally correct differential DNA extraction efficiencies and other biases in microbiome sequencing data, paving the road to cross-protocol comparisons in microbiome research.

## Methods

### Study design and sample preparation

#### Mock community samples and negative controls

**Fig. 1:**
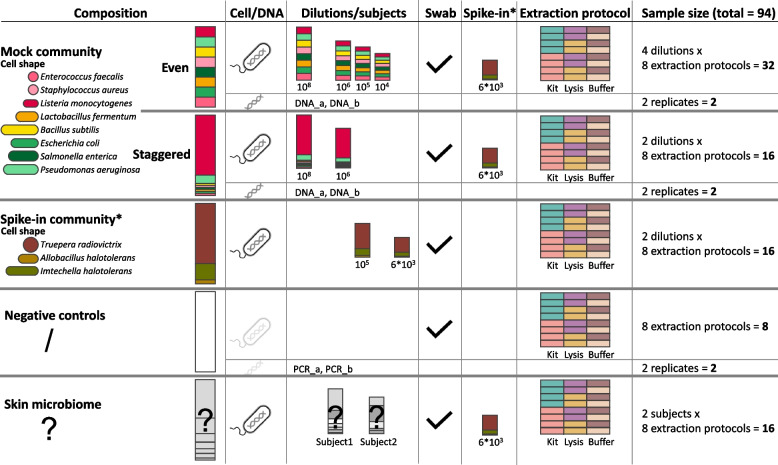
Study design of mock communities, controls, and skin microbiome samples to study extraction bias in eight extraction protocols. Cell-based mock communities with an even or a staggered composition diluted to 10^8^ to 10^4^ input cells and their corresponding DNA mocks were used to determine extraction bias. Eight different extraction protocols were tested in a combination of two extraction kits, two lysis protocols, and two extraction buffers. Additionally, negative controls and environmental skin microbiome swab samples underwent these eight extraction protocols. A swab was also added to all non-skin samples before DNA extraction. Asterisks (*) denote the spike-in community, of which 6 × 10^3^ cells were spiked into each mock and skin sample, and of which pure spike-in community samples were also investigated in two dilutions from 10^5^ to 6 × 10.^3^ input cells. Even and staggered DNA mock samples and PCR controls were processed in two replicates each (_a, _b)

An overview of our study design is shown in Fig. 1. We used mock microbial community standards with different taxa and abundance compositions provided by the ZymoBIOMICS series of ZymoResearch (Irvine, CA, USA). Two of these mock communities contain the eight bacterial species *Enterococcus faecalis*, *Staphylococcus aureus*, *Listeria monocytogenes*, *Lactobacillus fermentum* (also known as *Limosilactobacillus fermentum*), *Bacillus subtilis*, *Escherichia coli*, *Salmonella enterica*, and *Pseudomonas aeruginosa*, and the two fungal species *Saccharomyces cerevisiae* and *Cryptococcus neoformans*. Due to the lack of the 16S rRNA gene in fungi, the latter two species cannot be detected by our 16S rRNA gene sequencing approach and are therefore not further discussed. This eight-bacteria mock is available as a whole-cell mock community with an even (D6300) or staggered composition (D6310) and as corresponding DNA mocks with an even (D6305) or staggered (D6311) composition of DNA. The spike-in community (D6321) is only available as a staggered whole-cell community and consists of the three species *Truepera radiovictrix, Allobacillus halotolerans*, and *Imtechella halotolerans*, all of which are alien to the human microbiome. Table 1 shows the expected composition of all mock communities, considering 16S gene copy numbers and genome size for 16S rRNA gene sequencing experiments.

**Table 1:**
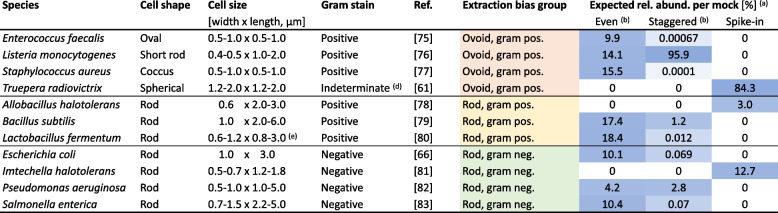
Based on cell shape and gram stain, bacterial taxa in our mock communities can be classified into three morphological groups that determine extraction bias in mock data [61, 66, 75–83]. Cell shape, cell size, and gram stain were extracted from scientific references (Ref.) and were used to divide all eleven mock taxa into three morphological groups. Red, green, and yellow background color highlight morphology-based groups of extraction bias, darker blue background color indicates higher expected relative abundance (rel. abund.) per mock community

^(a)^Expected relative abundances based on 16S theoretical composition provided by ZymoResearch (https://www.zymoresearch.de/collections/zymobiomics-microbial-community-standards), considering 16S copy numbers, genome size, genomic DNA content, and cell numbers per mock

^(b)^Depending on the dilution, the even and staggered mock community also contained varying proportions of spike-in taxa, which were not considered in bias calculations of the even and staggered mock community

^(d)^*T. radiovictrix* has indeterminate gram stain but has a thick multi-layered cell wall similar to gram-positive bacteria

^(e)^Information on cell size was only available for *L. reuteri*, a species of the *Lactobacillus* genus

The three cell mock communities (even, staggered, and spike-in) were tenfold or 20-fold diluted with Buffer AVE (Qiagen, Hilden, Germany) and split into eight replicates per mock and dilution for subsequent DNA extraction. The final mock bacterial input per sample ranged from 10^8^ to 10^4^ in the even mock, from 10^8^ to 10^6^ in the staggered mock, and from 1.1 × 10^5^ to 5.55 × 10^3^ cells (rounded to 10^5^ and 6 × 10^3^ cells for simplicity), as shown in Fig. 1. All 48 even and staggered cell mock samples were spiked with 5.55 × 10^3^ cells of the spike-in mock. The 64-cell mock samples (even, staggered, and spike-in) also contained empty swabs (DRYSWAB MWE, Cat No. MW 940/125, Medical Wire, Corsham/Wiltshire, UK), which are typically used in skin microbiome sampling, to compare with skin microbiome samples. Eight empty tubes containing only a swab were processed along with the samples as full-pipeline negative controls.

#### Skin microbiome samples

Skin microbiome samples were added in this study to test the feasibility and taxon recovery of different extraction approaches in skin microbiome research. Therefore, the skin microbiome of two healthy subjects was sampled by striking the forearm with eight parallel swabs (DRYSWAB MWE Cat No. MW 940/125, Medical Wire). Due to this parallel sampling approach, these skin samples may be subject to local variation in taxon relative abundances and thus do not represent true technical replicates. The 16 environmental skin microbiome samples were also spiked with 5.55 × 10^3^ cells of the spike-in mock. The collection of skin microbiome samples for methodological work was approved within the frame of the ProRaD study by the ethics committee of the Technical University of Munich (112/16S), and study subjects provided written informed consent for participation.

### Sample processing

#### DNA extraction

The eight replicates of each mock dilution and subject underwent eight different extraction protocols, representing a combination of two extraction kits, two lysis conditions, and two extraction buffers. We compared the extraction kits QIAamp UCP Pathogen Mini Kit (Cat No. 50214, Qiagen, Hilden, Germany; “Q”) versus ZymoBIOMICS DNA Microprep Kit (Cat No. D4301, ZymoResearch, Irvine, CA, USA, “Z”). The kit variable includes the beads used for cell lysis, with 0.1- and 0.5-mm Zirconia beads provided in the ZymoResearch kit (“Z”) and 0.1 µm Zirconia Beads (BioSpec, Bartlesville, Oklahoma) used for the Qiagen kit (“Q”). The two lysis conditions were a “soft” lysis condition (“S”) at 5600 RPM (rounds per minute) for 3 min versus a rather “tough” lysis condition (“T”) at 9000 RPM for 4 min, both on a Precellys Evolution Touch homogenizer (Bertin, Montigny-le-Bretonneux, France). Extraction buffers comprised DNA/RNA shield R1100-50 stabilizer (“z”) provided with the ZymoResearch kit, and a combination of Stool Stabilizer (Stratec, Birkenfeld, Germany) as preservative and Buffer ATL provided in the Qiagen kit (both summarized as “q”). Abbreviations per extraction protocol combination are used throughout this work in the format “kit_lysis_buffer”, e.g., “Q_T_z” for the combination of the Qiagen extraction kit, the tough lysis condition, and the ZymoResearch buffer.

All 88 whole-cell samples of diluted mock, full-pipeline negative controls, and skin microbiome with their respective buffers were frozen at − 80 °C before further processing, and only the Buffer ATL of “q” was added after thawing the samples for ensuing DNA extraction.

#### 16S rRNA gene amplification and sequencing

After DNA extraction, we added two replicates of even (D6305) and staggered (D6311) DNA mock community samples with 0.1 µl/ng DNA concentration (roughly equivalent to 1.3 × 10^7^ input cells), and two PCR negative controls, summing to a total of 94 samples. In the first PCR step, the V1–V3 variable region of the 16S rRNA gene was amplified using the Q5 Hot Start High-Fidelity DNA polymerase (New England Biolabs, Ipswich, MA, USA), and primers 27F-YM (5′-AGAGTTTGATYMTGGCTCAG-3′) and 534R (5′-ATTACCGCGGCTGCTGG-3′) with Illumina adaptor sequences. PCR conditions included an initial denaturation step at 98 °C for 1 min, followed by 25 cycles of 98 °C for 10 s, 59 °C for 20 s, and 72 °C for 15 s, and a final elongation step at 72 °C for 2 min. In a second PCR step, sample-specific dual-indexed barcodes were added to allow for multiplexed sequencing. The second PCR was run with an initial denaturation step at 98 °C for 40 s, followed by 8 cycles of 98 °C for 20 s, 55 °C for 40 s, and 72 °C for 40 s, and a final elongation step at 72 °C for 2 min. Indexed amplicons were purified twice using AMPure XP beads (Beckman Coulter, Fullerton, CA, USA) according to the manufacturer’s instructions and quantified using the fluorescent dye-based Qubit® dsDNA HS Assay Kit (Invitrogen, Carlsbad, CA, USA). Samples were equimolarly pooled and sequenced using the Illumina MiSeq platform (Illumina Inc., San Diego, CA, USA) with the MiSeq® Reagent Kit v3 (Illumina Inc.) to produce 2 × 300 bp reads.

### Data analysis

#### Raw sequence processing

High-quality reads according to Illumina specifications were de-multiplexed by their sample-specific index barcode sequences using the MiSeq® Reporter software. Raw sequences from FASTQ files were quality-controlled and denoised using DADA2 (version 1.16.0) [4]. Default filtering parameters were used, except for trimLeft = 20 to remove the forward primer and truncLen = 299, and nbases = 10^9^ for learning error rates in DADA2. Due to relatively low-quality reverse reads, we processed only the forward reads to keep the highest possible proportion of sequences for subsequent analysis. We did not remove chimeras or contaminants to allow for investigation of chimera formation and protocol-dependent contaminants.

#### Taxonomic classification

Taxonomic annotation of the resulting amplicon sequence variants (ASVs) was performed by comparison against the reference 16S rRNA gene sequences provided by ZymoResearch (available from https://s3.amazonaws.com/zymo-files/BioPool/ZymoBIOMICS.STD.refseq.v2.zip). Levenshtein distance (edit distance, LV) was calculated between expected sequences (cut to 279 bp) and observed ASV sequences, with LV = 0 indicating an exact match with the reference sequence. We used the smallest LV distance to any expected sequence as a proxy for the number of sequence errors (substitutions or indels) introduced during amplification or sequencing. Ambiguous LV annotations between *E. coli* and *S. enterica* were resolved for 6 ASV sequences with LV ≤ 8 (maximum relative abundance 2.3%) using the DNA evolution model [36]. ASV sequences with LV > 8 to any expected sequence (corresponding to < 97% identity) were further checked for their longest common substring (LCS) with the 279-bp-long expected reference sequences. When > 95% (266 bp) of the sequence exactly matched with parts of two or three different mock taxa, we interpreted these sequences to represent chimeras between mock taxa. All remaining ASVs that could not be explained by small LV distance or a mixture of exact matches were defined as “unclassified”, representing mostly skin and contaminant taxa, but potentially also chimeras with ≥ 4 parts or chimeras between mock and skin/contaminant taxa.

Additionally, we used the RDP- and NCBI-based annotation provided through DADA2 [37] to get taxonomic information on species not part of the ZymoResearch mock communities, such as the skin taxa.

#### Statistical analysis

All statistical analyses and visualizations were created using R (version 4.0.2) [38]. The R packages “tidyverse” (version 2.0.0) [39], “reshape2” (version 1.4.4) [40], “ComplexHeatmap” (version 2.4.3) [41], “rstatix” (version 0.7.2) [42], “ggh4x” (version 0.2.4) [43], “ggConvexHull” (version 0.1.0) [44], “ggpubr” (version 0.6.0) [45], and “cowplot” (version 1.1.1) [46] were used for data preparation and visualization. Hierarchical clustering of heatmaps was performed using Euclidean distance and complete linkage. The clustering of “unclassified” ASVs was done by k-means implemented in R with default parameters and 4 clusters. LV distance was calculated using the R package “stringdist” (version 0.9.10) [47], the DNA evolution model using the package “ape” (version 5.7–1) [48] and the longest common substring (LCS) using the package “PTXQC” (version 1.0.16) [49].

ASVs with LV ≤ 4 to any expected sequence were accepted to represent mock taxa and were summarized to species level for subsequent analyses. Similarly, DADA2-based taxonomic annotation was summarized to genus or species level for all analyses of the skin samples and contaminant ASVs. Spearman’s correlation coefficient *r*_S_ was used to measure associations between continuous variables. When calculating distances between samples, we used the traditional Bray–Curtis dissimilarity and the compositional Aitchison distance. For all analyses using Bray–Curtis dissimilarities, reads were transformed to relative abundances by total sum scaling (TSS). For all analyses using Aitchison distance or logarithm, zero counts were replaced by 0.5 and zero relative abundances by 0.00001. For beta diversity analyses of mock and spike-in samples, only mock taxa were considered. Bray–Curtis or Aitchison distances were visualized using principal coordinate analysis (PCoA) provided through wcmdscale() of the r package “vegan” (version 2.6–4) [50], and corresponding p-values were calculated using PERMANOVA.

To calculate bias per taxon and protocol, we applied the metacal approach [1] implemented in the R package metacal (version 0.2.0) [35]. Metacal represents a compositional mathematical model, that assumes microbiome sequencing data to be a multiplicative result of input relative abundance and taxon-specific biases and allows for calculating and correcting these biases. In the staggered mock community, only the four most abundant taxa (with expected relative abundance ≥ 0.07%, see Table 1) were consistently detected across the staggered cell and DNA mock samples. Since Aitchison distance is sensitive to changes in small count values, we only considered these four consistently detected taxa in all bias calculations and corrections. For the same reason, we excluded zero counts from all metacal-based analyses in one 10^4^ sample of the even mock community and in one 6 × 10^3^ sample of the spike-in community, and completely excluded two spike-in samples where none or only one of the spike-in taxa was detected. When correcting extraction bias by bacterial morphology, we followed the approach described by McLaren et al. [1] and divided observed relative abundances per sample by the extraction bias values calculated from the 10^6^ even mock training sample. Therefore, extraction bias per taxon was summarized by its geometric mean per morphology group. Due to mixed results for bias correction in the Z_T_q/z protocols in mock samples, we excluded these protocols when showing the impact of the bias correction in the skin samples.

For all analyses using randomness, set.seed(1) was run for reproducibility. Wilcoxon signed-rank test was used to assess differences in extraction bias between morphology groups and the reduction of bias after computational correction.

## Results

### Sequence classification and sequence errors

**Fig. 2:**
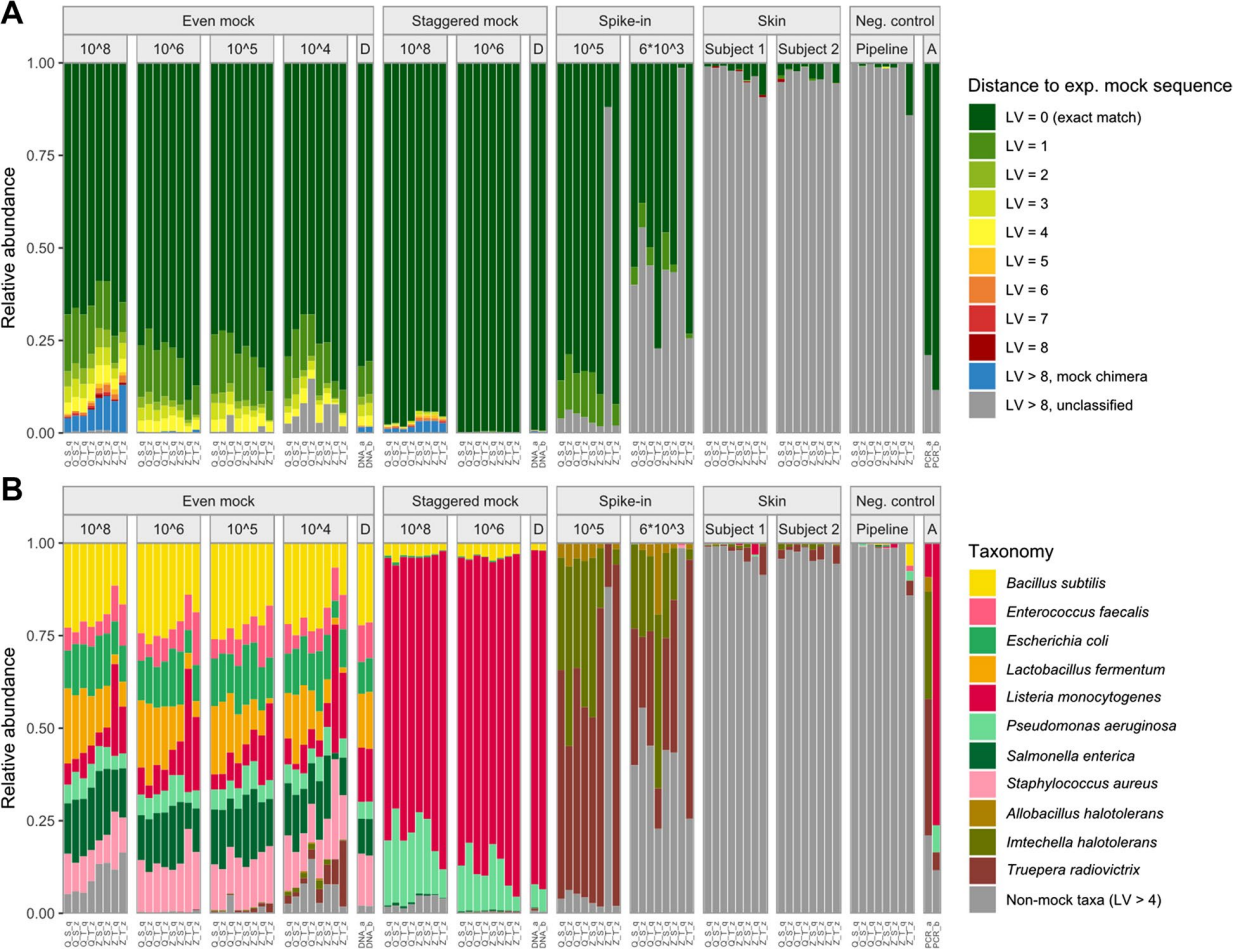
Taxonomic annotation by comparison to expected sequences reveals sequence errors, chimeras, contamination, and differences in sample composition between extraction protocols. Levenshtein (LV) distance between observed ASV sequences and expected mock sequences allows for classifying sequences into exact matches, sequence errors, chimeras, and remaining “unclassified” sequences with LV > 8 (**A**). After accepting sequence errors with LV ≤ 4 as correct ASVs, considerable differences in sample compositions between extraction protocols were found, but also cross-contamination from mock into the skin and negative control samples (**B**). Chimeras (**A**) were defined as sequences with LV > 8 to any expected sequences and > 95% identity by LCS with at least two expected mock sequences. Sample composition (**B**) is shown for the 11 expected mock taxa, with non-mock taxa (LV > 4) representing, e.g., contaminants and skin taxa. Q: Qiagen extraction kit, Z: ZymoResearch extraction kit, S: “soft” lysis condition, T: “tough” lysis condition, q: Qiagen/Stratec buffer, z: ZymoResearch buffer. A: Amplification/PCR control. D: DNA mock samples

We prepared a dilutions series of three commercially available mock communities from ZymoResearch. These mocks were used together with negative controls and environmental skin samples to study eight different extraction protocols in a combination of two extraction buffers, two lysis protocols, and two extraction kits. The resulting ASVs from V1–﻿V3 16S rRNA gene sequencing were taxonomically annotated by matching against reference sequences provided by ZymoResearch. This taxonomic matching was done by combining LV distance with LCS, which allowed for classifying ASVs into exact matches, sequence errors, chimeras between mock sequences, and remaining “unclassified” sequences (Fig. 2A).

The highest proportion of correct sequences was observed in the staggered mock, reaching 99.7% (median) of exact matches in samples with 10^6^ input cells. All even mock samples presented with considerably larger proportions of sequence errors (range 6.2% to 31.6%, median 22.3%). Interestingly, most sequence error ASVs were only present in the 10^8^ input samples. In contrast, the majority of sequence error reads in even mock samples were assigned to 8 ASVs of *L. fermentum*, *E. coli*, or *S. enterica* (76.1%) and were consistently detected across all even mock samples (Supplementary Figure S1). These three species jointly make up only 0.15% of the expected staggered mock composition, explaining the lower proportion of sequence errors observed in the staggered mock.

Due to the ubiquitous presence of sequence errors with LV ≤ 4 in our data (Fig. 2A), we accepted these sequences to represent valid mock taxa for all subsequent analyses. The resulting final taxonomic distribution of mock and spike-in taxa over samples is shown in Fig. 2B.

### Chimera formation

**Fig. 3:**
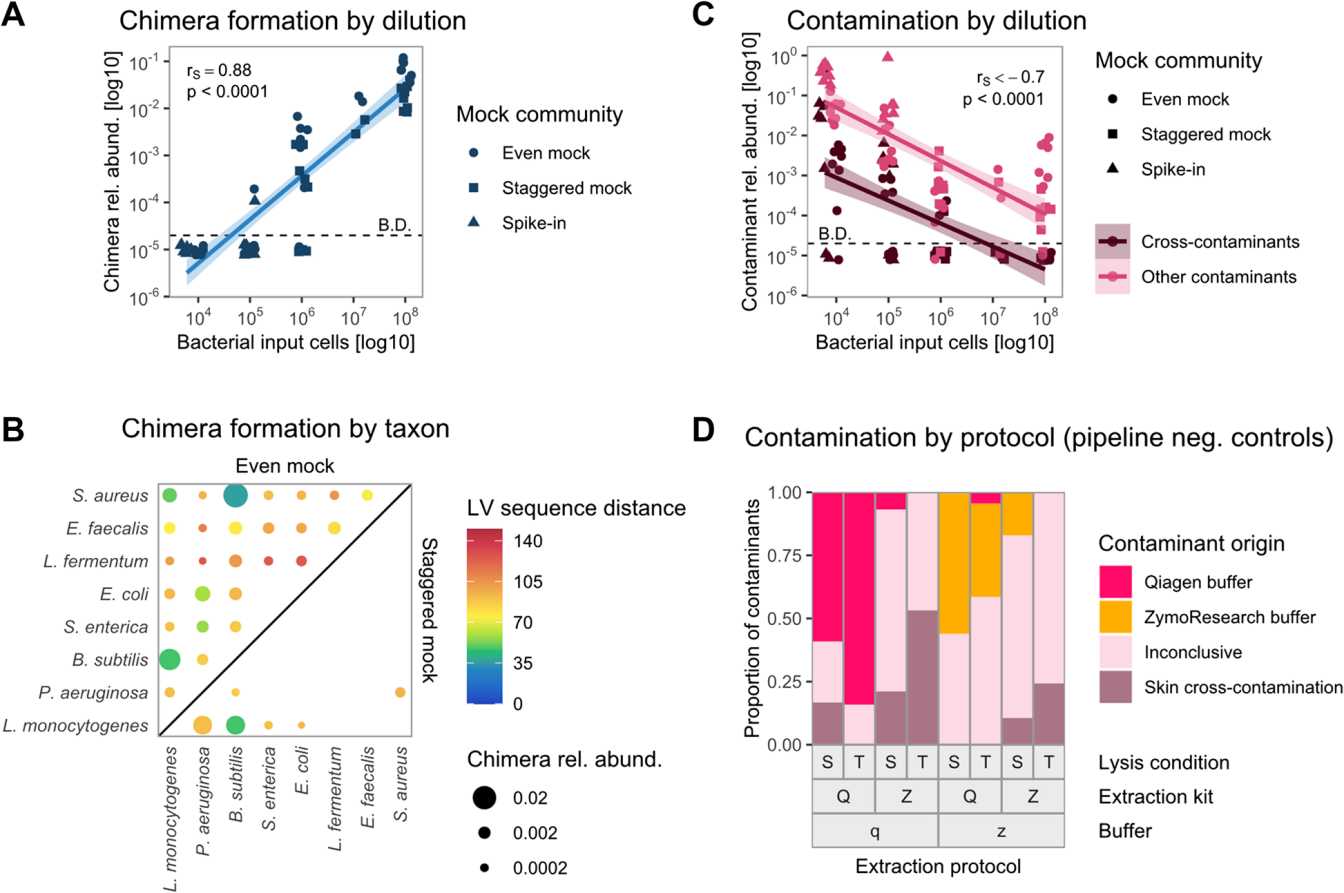
Chimera formation between closely related and highly abundant species increases with high bacterial input, and contamination decreases with bacterial input and originates mostly from buffers. Chimera relative abundance is significantly positively correlated with bacterial input cells per sample across all mock communities (**A**). Chimeras were predominantly formed between closely related species in the even mock (upper left diagonal), indicated by blue/green color, or between highly abundant species in the staggered mock (lower right diagonal), as indicated by species order in decreasing expected abundance from left to right (**B**). Both cross-contaminants and other contaminants significantly negatively correlated with bacterial input cells per sample across all mock communities (**C**). Contamination in pipeline negative controls was substantially associated with extraction protocols, with distinct contaminants originating from the two extraction buffers (**D**). Zeros (**A**, **C**) were replaced by 0.00001, indicated as below detection (B.D.). Correlations were estimated by Spearman's rank correlation coefficient rho (*r*_S_). Points in **A** and **C** are jittered for better visibility, and lines in **A** and **C** represent regression lines based on linear models with 95% confidence intervals. Chimera pairs (**B**) are only shown for 10^8^ input cell samples, and the point area indicates chimera relative abundance per sample. Chimeras (LV > 8) between *E. coli* and *S. enterica* could not be identified due to their small sequence distance (minimum LV = 6). Color represents the mean LV distance between 16S rRNA copy variants of two taxa, based on reference sequences provided by ZymoResearch and cut to 279 bp of the V1-V3 region. Contaminant categories (**D**) were determined by kmeans clustering of ASV relative abundances over all mock and control samples, relative abundance per contaminant origin is shown out of total contamination per sample. LV: Levenshtein, Rel. abund.: relative abundance, Q: Qiagen extraction kit, Z: ZymoResearch extraction kit, S: “soft” lysis condition, T: “tough” lysis condition, q: Qiagen/Stratec buffer, z: ZymoResearch buffer

Surprisingly, chimera formation increased with higher sample biomass, reaching up to 13% in samples with 10^8^ input cells (Fig. 2A). Samples with ≤ 10^5^ input cells contained < 0.026% chimeras, and the proportion of chimeric reads was significantly positively correlated with the number of input cells per sample (*r*_S_ = 0.88, *p* < 0.001, Fig. 3A).

Investigating chimera formation in more detail, we found that most chimeric combinations were consistently detected across protocols (Supplementary Figure S2). Many three-part chimeras (“trimeras”) were actually composed of only two different species, interrupting the exact matching of the LCS with short sequence errors. Therefore, we focused only on the two unique species with the longest LCS per chimera for subsequent analyses, and on samples with 10^8^ input cells due to their high relative abundance of chimeras. Chimeras in the even mock were overall more diverse, and all discernible taxon combinations were detected (Fig. 3B). However, most chimeric reads were formed by pairs of *S. aureus*, *B. subtilis*, and *L. monocytogenes*, or of *P. aeruginosa* and *E. coli*., reflecting the groups of taxa with high sequence similarity (Fig. 3B, Supplementary Figure S3). In the staggered mock, we observed overall less chimera formation and fewer combinations of taxa, with chimeras mainly formed between the most abundant species *L. monocytogenes*, *B. subtilis*, and *P. aeruginosa* (Fig. 3B). Taken together, our data indicate that higher microbial DNA input leads to increased chimera formation between closely related and highly abundant sequences.

### Contamination and cross-contamination

Apart from expected sequences, sequence errors, and chimeras, the proportion of remaining “unclassified” ASVs was significantly negatively correlated with the amount of bacterial input cell numbers per sample (*r*_S_ <  − 0.7, *p* < 0.0001, Fig. 3C). We further investigated these presumably contaminating “unclassified” ASVs present in at least three mock and control samples using kmeans clustering of their relative abundances per sample. A few specific ASVs of these “unclassified” reads originated from the two extraction buffers (Supplementary Figure S4). Using the RDP-based annotation, contaminants present in the Stratec/Qiagen buffer (“q”) were assigned to *Methylobacterium*, *Alcaligenes*, and *Brucella*, whereas contaminants present in the ZymoResearch buffer (“z”) belonged to *Paraburkholderia*, *Aquabacterium*, *Nitrospirillum*, and *Herbaspirillum*. Another group of two clustered ASVs was assigned to *Cutibacterium* and *Pseudomonas*. Together with other *Staphylococcus* and *Corynebacterium* ASVs in the fourth cluster of “inconclusive” origin, these genera represent major taxa present in our skin microbiome samples, indicating contamination from lab operators or cross-contamination from the skin samples. Figure 3D shows the substantial proportion (median 47.3%) of both buffer contaminants and skin cross-contaminants among all “unclassified” reads detected in negative controls.

Apart from the above-mentioned skin taxa and small proportions of spike-in taxa intentionally spiked into the skin samples, we also sporadically detected mock taxa in skin samples. Therefore, we observed not only skin-to-mock contamination but also mock-to-skin and mock-to-control contamination (Fig. 2A, B), clearly supporting cross-contamination in our data. Comparable to contaminants, the proportion of cross-contaminants per sample was significantly negatively correlated with mock bacterial input (*r*_S_ =  − 0.70, *p* < 0.0001) and increased with lower bacterial input cell numbers (Fig. 3C).

### Effect of extraction protocols

Having resolved the origin of sequence errors, chimeras, and contaminants, we investigated the effect of extraction protocols on global sample composition, focusing only on each sample’s expected taxa with LV ≤ 4 in the following beta diversity analyses.

**Fig. 4:**
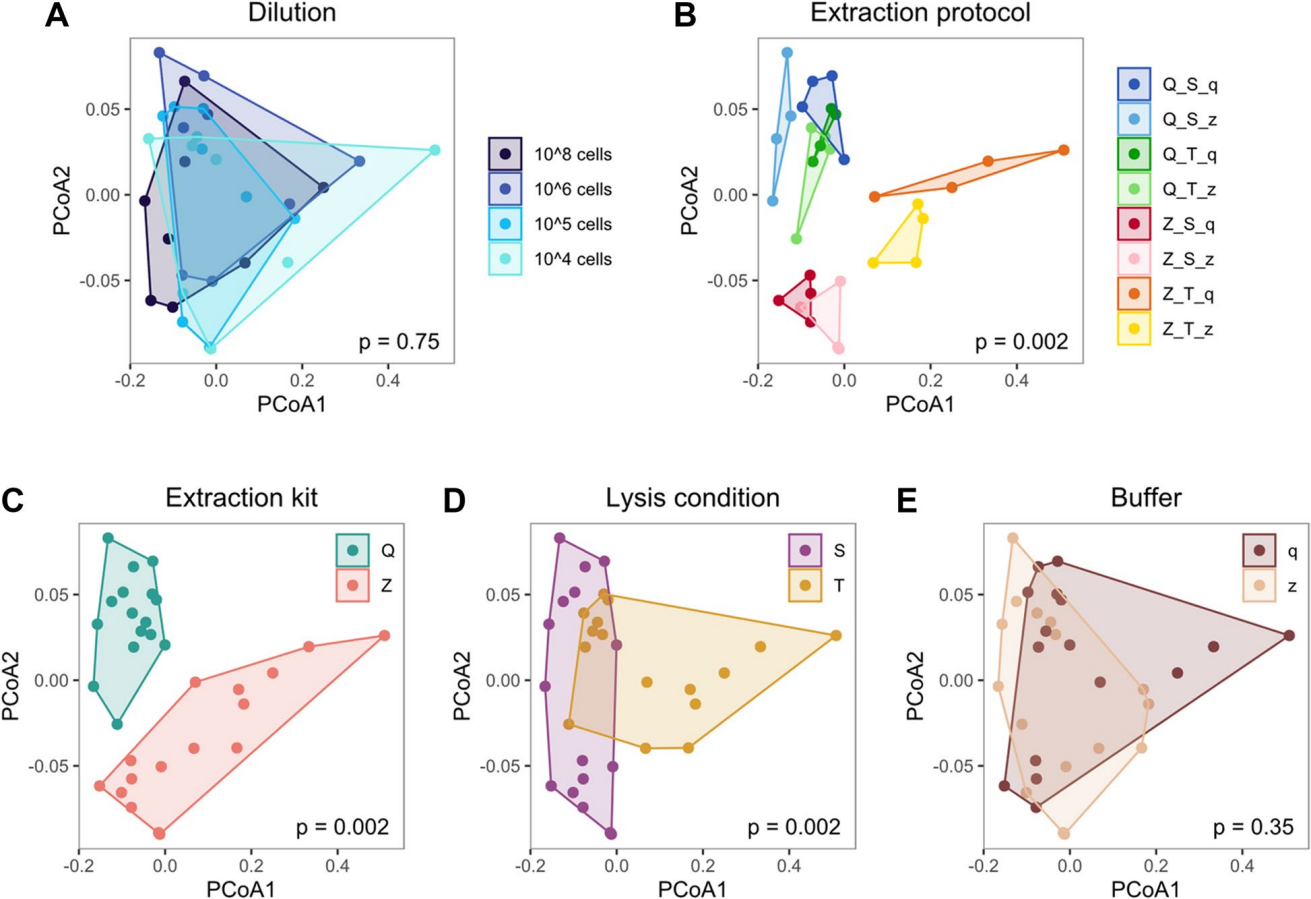
Sample composition is significantly affected by extraction protocols, particularly by extraction kit and lysis condition in the even mock community. Beta diversity analysis revealed significant differences in global mock composition between extraction protocols (**B**), kits (**C**), and lysis conditions (**D**), but not between dilutions (**A**) or buffers (**E**). Beta diversity was performed only on mock taxa with LV ≤ 4 to any expected mock sequence, and is visualized by PCoA on Bray–Curtis dissimilarities. Polygonal shaded areas connect samples of the same group, *p*-values are derived from PERMANOVA tests with 500 permutations. Q: Qiagen extraction kit, Z: ZymoResearch extraction kit, S: “soft” lysis condition, T: “tough” lysis condition, q: Qiagen/Stratec buffer, z: ZymoResearch buffer

Considering only the eight expected bacteria in the even mock, we found no significant difference in sample composition between dilutions (*p* = 0.75, Fig. 4A). Therefore, we treated dilutions as replicates for studying protocol effects (Fig. 4B–E). The choice of our eight extraction protocols had substantial effects on sample composition, creating eight almost distinct groups of samples (*p* = 0.0020, Fig. 4B). When comparing extraction protocol details, we found significant differences between extraction kits (*p* = 0.0020, Fig. 4C) and lysis conditions (*p* = 0.0020, Fig. 4D). Only the two extraction buffers led to comparable sample compositions (*p* = 0.35, Fig. 4E).

Analysis of the staggered mock (considering eight mock taxa, Supplementary Figure S5A–E) and spike-in samples (considering three spike-in taxa, Supplementary Figure S5F–J) overall confirmed the results of the even mock. We found significant differences in sample composition between lysis conditions in the staggered mock (*p* = 0.008), and in the spike-in samples between all eight extraction protocols (*p* = 0.012) and between extraction kits (*p* = 0.012). These larger and less conclusive p-values might be explained by the smaller sample size of only two instead of four dilutions per sample group.

Based on the aforementioned results, we removed buffer contaminants and cross-contaminants of mock origin from the skin microbiome samples. Interestingly, the skin samples differed significantly only between the two subjects (*p* = 0.006, Supplementary Figure S5K). Apparently, differences in individual skin genera were so pronounced that they masked any other effect of extraction protocols in the skin samples (*p* > 0.098, Supplementary Figure S5L–O). Overall, these beta-diversity results based on Bray–Curtis dissimilarities were similar to those obtained using Aitchison distance.

### Which extraction protocol is best?

With substantial differences observed in sample composition between protocols, we aimed to determine the best extraction protocol. Therefore, we compared the sample composition of each cell sample to their corresponding DNA mock samples, to only evaluate extraction bias and not bias our analyses, e.g., by differential amplification bias between taxa. Again, we focused only on the eight (even, staggered) bacterial taxa per mock community.

With lower distances and lower ranks indicating less extraction bias (Supplementary Figure S6A, B), none of the protocols achieved a perfect representation of the expected DNA mock-based sample composition. Interestingly, although the choice of buffer did not significantly affect the sample composition, we found that each extraction kit consistently produced less bias when combined with its corresponding extraction buffer (Supplementary Figure S6C). Again, input cell dilutions and the choice of Bray–Curtis dissimilarity or Aitchison distance measure led to very similar results.

In contrast, the performance of extraction protocols was very distinct between the even and the staggered mock community. Although good results were achieved for example with the Q_T_q and the Z_T_z protocol in both mock communities, it was impossible to determine a single best extraction protocol across both mocks. But even more importantly, since each sample’s total extraction bias varied between identically processed but dissimilarly composed samples, it seemed that extraction bias does not only differ between protocols but also between taxa.

### Morphology-based correction of extraction bias

To follow up the hypothesis of taxon-specific, protocol-dependent extraction bias, we used the compositional metacal approach [1] to calculate extraction bias per species and protocol. Bias in cell mocks was calculated per sample and compared to corresponding DNA mocks for the even and staggered mock and to the expected composition for the spike-in mock community.

**Fig. 5:**
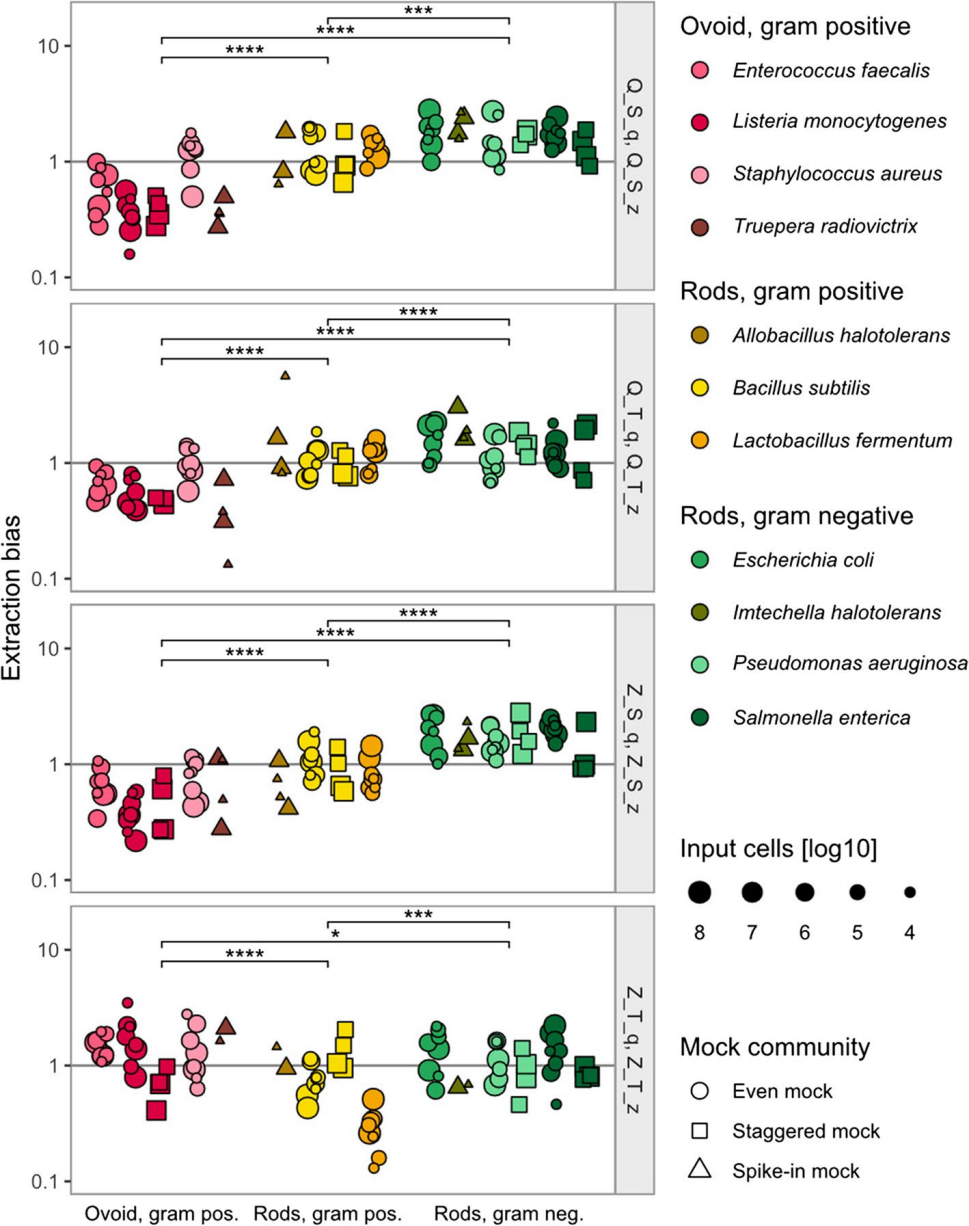
Extraction bias per protocol is significantly different between morphology-based groups of bacteria over different mock communities and input cell numbers. Morphological groups of extraction bias were formed based on cell shape and gram stain. Using the metacal R package, extraction bias per sample was calculated in relation to the respective DNA mock composition (even, staggered mock) or to the expected mock composition (spike-in mock). Statistical significance in extraction bias between morphology groups was assessed using Wilcoxon signed-rank test with Benjamini–Hochberg *p*-value adjustment, with *p < 0.05, ***p < 0.001, ****p < 0.0001. The point area indicates the number of input cells per sample, points are jittered for better visibility, and the vertical line represents no extraction bias. Gram pos.: gram-positive, gram neg: gram negative, Q: Qiagen extraction kit, Z: ZymoResearch extraction kit, S: “soft” lysis condition, T: “﻿tough﻿” lysis condition, q: Qiagen/Stratec buffer, z: ZymoResearch buffer

As hypothesized, we indeed found that extraction bias profiles varied substantially between species (Fig. 5). However, some groups of species also presented very similar extraction bias profiles over the eight extraction protocols, and even over different mock or spike-in sample compositions. We found that these groups were distinctively defined by the bacteria’s cell shape and cell wall structure (Table 1). Due to the small set of only eleven species, we were not able to distinguish if the effect of cell shape is actually rather an effect of cell length or size, and continue using the terms “shape” and “gram stain” for simplicity. Generally, we found that ovoid gram-positive bacteria were underrepresented (protocols Q_S_q/z, Q_T_q/z, Z_S_q/z), whereas rod-shaped gram-negative bacteria were easier to lyse and rather overrepresented. This pattern only turned when using the Z_T_q/z protocols, with ovoid gram-positive bacteria being slightly overrepresented and rod-shaped bacteria being slightly underrepresented. Of note, the Z_T_q/z protocols seemed to be less affected by systematic extraction bias between morphology groups but resulted in considerably low reads and missing expected taxa in the spike-in and skin samples, particularly when combined with the “q” buffer (data not shown). Overall, bacterial cell morphology clearly seemed to determine protocol-specific extraction bias independent of the sample composition.

Next, we tested if these protocol- and morphology-based bias profiles can be used for correcting extraction bias. Therefore, we calculated the extraction biases for the eight expected species and eight protocols, but using only a single sample per extraction protocol (10^6^ even cell mock, corresponding to “training data”, based on high sequence accuracy determined in previous sections). We summarized these 64 bias values by our morphology groups, leading to 24 correction factors (8 protocols × 3 morphology groups) shown in Supplementary Table S1. These factors were then applied to correct other samples (corresponding to “validation data”). The success of bias correction was evaluated as a decrease in the distance to the DNA (even, staggered mock) or expected mock composition (spike-in mock).

**Fig. 6:**
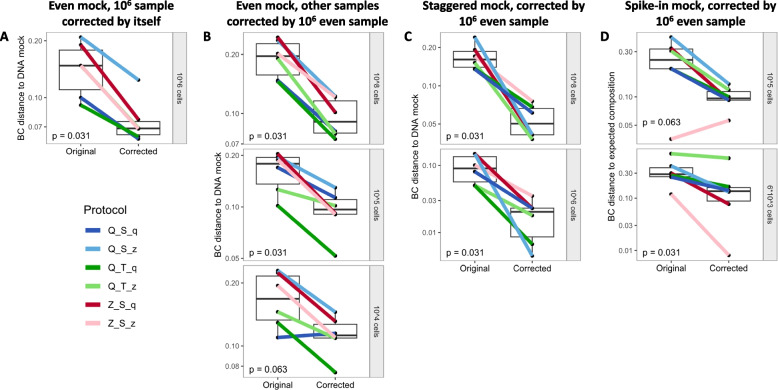
Distance to the DNA or expected mock composition is reduced after applying morphology-based correction of extraction bias, shown for six extraction protocols. Extraction bias per protocol was calculated from the 10^6^ even mock samples, summarized by bacterial morphology group, and applied to the 10^6^ even mock samples (internal correction, **A**), but also to different samples of the same mock (even mock 10^8^, 10^5^, 10^4^ cells, **B**), different samples of a different mock (staggered mock, **C**), and to different samples with different taxa (spike-in mock, **D**). Only the six protocols are shown here, where extraction bias was clearly separated by bacterial morphology (Q_S_q/z, Q_T_q/z, and Z_S_q/z, see Fig. 5), whereas all protocols are shown in Supplementary Figure S7. Extraction bias was measured by Bray–Curtis dissimilarity to the DNA mock composition (even mock, staggered mock) or to the expected mock composition (spike-in mock). Boxes denote the median and interquartile range (IQR), whiskers represent values up to 1.5 times the IQR, and dots indicate individual samples. Q: Qiagen extraction kit, Z: ZymoResearch extraction kit, S: “soft” lysis condition, T: “tough” lysis condition, q: Qiagen/Stratec buffer, z: ZymoResearch buffer

Overall, extraction bias (measured by Bray–Curtis dissimilarity) was reduced by this bias correction approach across different mock compositions and dilutions (Supplementary Figure S7). However, since the extraction bias in the Z_T_q/z protocols was less separated between the morphology groups (Fig. 5), we tested the statistical significance of the bias correction also without the Z_T_q/z protocols (Fig. 6). A significant bias reduction was achieved when correcting the 10^6^ even sample (*p* = 0.031, Fig. 6A, internal correction of the training sample), but also when correcting other dilutions of the even mock not involved in the bias calculation, except for the lowest dilution at 10^4^ cells (*p* = 0.031 for 10^8^ cells, *p* = 0.031 for 10^5^ cells, *p* = 0.063 for 10^4^ cells, Fig. 6B).

Next, we used the morphology-based bias factors of the 10^6^ even training sample to test the correction of a different sample composition in the staggered mock. Again, extraction bias was significantly reduced for both dilutions over the six protocols (*p* = 0.031 for 10^8^ and 10^6^ cells, Fig. 6C).

As a last test, we applied the morphology-based bias correction from the 10^6^ even mock training samples to the spike-in samples, which represent different samples, with different sample compositions, and even with completely different species as compared to the training sample. Here, the median distance to the expected composition was again reduced in both dilutions (*p* = 0.063 for 10^5^ cells, *p* = 0.031 for 10^3^ cells, Fig. 6D). Results evaluating the bias reduction using Aitchison distance to the DNA or expected compositions were overall comparable.

Taken together, the morphology-based correction of extraction bias achieved a median bias reduction of 57% in the spike-in samples (without Z_T_q/z protocols), measured as a decrease in median Bray–Curtis dissimilarities from 0.29 to 0.11 compared to the expected composition. In the even and staggered mock, extraction bias was reduced by 51% from median Bray–Curtis dissimilarities of 0.15 to 0.075 compared to their respective DNA composition. Thus, after correcting extraction bias, the remaining dissimilarity to the DNA composition in the even and staggered mock is approximately equal to the median dissimilarity of 0.072 between the original mock dilutions per protocol. The median dissimilarity of the DNA mock samples to their respective expected mock compositions remains at 0.049.

In order to further assess the impact of our extraction bias correction model, we applied the correction factors derived from the 10^6^ even mock sample (Supplementary Table S1) to the skin samples included in our study (excluding Z_T_q/z protocols). Due to the absence of ground-truth information in these environmental microbiome samples, we could not determine the reduction in bias but only assess the impact on taxon relative abundances after applying the bias correction. Moreover, due to the parallel swabbing used for collecting the skin samples, we could not ensure that samples were true technical replicates, which does not allow for determining an improvement in sample similarity after the bias correction. However, we observed considerable changes in the overall sample composition on the genus level (Supplementary Figure S8A). When examining the top three skin species in more detail, changes ranged from a 29% decrease (0.71-fold) up to a 143% increase (2.43-fold) in their relative abundances (Supplementary Figure S8B).

## Discussion

Microbiome sequencing data are distorted by errors and protocol-dependent biases, confounding biological interpretations. Using dilution series of well-characterized mock communities, we investigated and removed sequence errors, chimeras, contaminants, and cross-contaminants. With the resulting precise species’ relative abundances, we propose novel associations between extraction bias and bacterial cell morphology, which we used to correct extraction bias computationally. This correction method reduced extraction bias in mock samples by half and showed a considerable impact on bacterial compositions when applied to environmental skin microbiome samples.

Samples of the even mock community were characterized by particularly high proportions of sequence errors, i.e., ASVs with close genetic distance to expected sequences. The consistent and highly abundant presence of a few major sequence error ASVs across replicates would suggest they are true minor 16S gene variants not covered by the reference genomes. However, consistent detection does not prove sequence validity [7]. Instead, these ASVs may represent deviations from actual sequences that are particularly susceptible to error formation [51] and are not corrected by our denoising approach using DADA2 [9]. Interestingly, the presence of both sequence errors and chimeras increased with higher bacterial cell numbers. Assuming that higher cell numbers lead to higher DNA concentration and thus to closer physical proximity between similar DNA motifs from different species, high bacterial input would promote chimera formation by template switch during PCR amplification [52]. In line with that, chimera formation occurred mainly between closely related species (even mock) and, to a smaller extent, between highly abundant species (staggered mock), as observed in our data and previous research [7]. It has been established that high DNA input material can inhibit the PCR and lead to unspecific amplification and that high numbers of PCR cycles should be avoided to reduce chimera formation [2, 52, 53]. However, with substantial chimera formation starting at 10^8^ bacterial input cells in our data, chimeras may hamper taxonomic annotation and diversity estimation particularly in microbiome samples from high-biomass environments, such as stool [54].

In contrast to chimeras, the proportion of contaminants and cross-contaminants increased with lower bacterial input material in our study. The threat of contamination to low-biomass samples is well-known [10, 14, 15], yet internal cross-contamination between samples is rarely investigated [11]. Our mock samples contained several skin-associated genera like *Cutibacterium*, *Pseudomonas*, *Staphylococcus*, and *Corynebacterium* [13], which however have also been identified as common contaminants in microbiome sequencing experiments [14], e.g., originating from lab operators [10]. We also detected mock taxa in skin and negative control samples and found spike-in taxa (alien to the human microbiome and lab reagents [14]) in negative controls. Therefore, we conclude that our low-biomass samples are subject to considerable cross-contamination, which would have been inseparable from external contamination in environmental microbiome studies.

A major source of external contamination in low-biomass samples are bacterial DNA extraction kits, which have been previously identified to harbor a specific “kitome” [10, 55]. Interestingly, most external contaminants in our data were not associated with extraction kits but with buffers. A previous investigation of contaminants in extraction kit components did not report buffer-specific contamination profiles but did not explicitly investigate external, non-mock taxa [56]. Validation of our findings would provide a simple roadmap to notably reduce contamination by treating only extraction buffers, e.g., with UV irradiation [57].

The two extraction buffers in our study led to distinct contamination profiles, but apart from this, the buffers did not significantly alter the global sample composition of expected mock taxa, despite giving slightly less biased sample compositions when combined with their corresponding extraction kits. In contrast, the choice of extraction kit and lysis condition (speed and duration) significantly affected the relative abundances of mock taxa in our data. Although these differences were smaller than inter-individual differences in the skin microbiome, observed here as well as in previous microbiome surveys [18, 27, 29, 31, 58, 59], extraction protocols still significantly contribute to the bias observed in microbiome sequencing data. Numerous studies have addressed this problem by benchmarking DNA extraction methods [18, 20, 27, 28], usually aiming at determining the best extraction protocol for specific microbiome environments [25, 26, 29, 60]. In support of this research direction, we demonstrated that choosing the best protocol with the least extraction bias depends on the sample composition and taxa of interest. Therefore, comparisons between microbiome studies remain challenging, and it may be impossible to agree on a single standard microbiome laboratory protocol in the scientific community.

In our study, no extraction protocol achieved perfect accuracy, each leading to more or less distorted sample compositions. Besides improving laboratory extraction methods, extraction bias could be resolved by a computational correction using defined bacterial mock communities as positive controls in each sequencing run. Here, we provide novel evidence for such a computational correction by linking extraction bias per protocol and taxon to bacterial cell morphology, specifically gram stain and cell shape. It is well-established that gram-positive bacteria are harder to lyse than gram-negative bacteria [24, 27, 31], demanding a mechanical lysis step such as bead-beating to lyse the thick gram-positive cell wall [19, 24, 28]. In the context of cell lysis, gram stain only serves as a proxy for cell wall thickness. For example, *Truepera radiovictrix* cannot be classified into gram-positive or gram-negative stains but possesses a thick three-layered cell wall [61] that led to extraction efficiencies similar to other gram-positive bacteria. Similarly, the effect of cell shape on extraction bias could have been equally represented by bacterial cell size. Bacterial cell size however depends on multiple environmental conditions and the measurement method chosen [62]. Indeed, we found large variability in reported cell sizes, e.g., for *L. monocytogenes*, ranging from 0.4 × 0.7 µm [63] up to 2 × 4 µm [64], or *E. coli*, ranging from 0.5 × 1 µm [65] up to 1 × 3 µm [66]. In lack of support for either hypothesis between cell shape or size, we propose that rod-shaped (= larger) bacteria are more easily hit by beads during mechanical lysis. In line with that, gram-negative and rod-shaped bacteria seemed to be rather overrepresented compared to gram-positive or ovoid bacteria, and the tougher protocol (“T”) slightly enhanced the relative abundance of gram-positive cocci.

Using the morphology-based computational correction, we significantly reduced extraction bias in new samples and even in new taxa using only gram stain and cell shape. Additional factors, such as aerobic status, motility, or endospore formation, may affect cell stability in the lab and could have improved our bias correction model. However, we were limited to a sparse set of bacterial properties in our 8- and 3-species mock communities. Interestingly, previous research has claimed that bias cannot be summarized for groups of taxa [1], supported by differential biases observed between species of the same genus [1, 2, 67]. However, the mock experiment by Brooks et al. [68] used species without available reference genomes, leading to ambiguous estimations of 16S copy numbers [1, 68] and potential problems with taxonomic annotation, which jointly blur precise abundance estimations. The experimental design by Morgan et al. [67] did not allow for specifically measuring extraction bias. Thus, differential amplification may have affected the bias of closely related species [1, 68]. We believe that only our combination of well-characterized mock communities with reference genomes, rigorous study design including corresponding DNA mocks, and in-depth analyses of downstream biases allowed for precisely assessing the relations between bacterial cell morphology and extraction bias.

Despite significantly reducing overall bias, both extraction and total bias were not completely eliminated. Since the cell and DNA mocks used in this study were designed to contain the same amount of genetic material per taxon in both mock types, most biases should equally affect cell- and DNA-based mock taxon compositions. Therefore, many systematic biases central to 16S-based microbiome studies [2] can be ignored here, such as differential primer binding, 16S copy numbers, or amplification bias. While these biases may explain the bias between our DNA mock samples and their expected compositions, they should not affect our analysis of extraction bias. Additionally, we attempted to account as much as possible for contamination, cross-contamination, sequence errors, chimeras, and annotation mistakes by matching observed sequences with mock reference genomes. However, DNA-based biases and the remaining extraction bias could originate from random errors during pipetting and aliquoting and from random events during sample storage, extraction, amplification, and sequencing [1, 69]. The impact of random effects is supported by the fact that the remaining distance of corrected cell mock samples to the DNA mock samples was approximately equal to the dissimilarities between mock dilutions.

Yet, a few limitations of our study should be noted. With outliers observed both in some species and some protocols, our approach requires potential refinement and validation in a larger mock study, allowing for better disentangling of the relations of additional bacterial properties and extraction bias. Currently, such extensive information on bacterial characteristics is difficult to obtain. Additionally, the study of bacterial characteristics is currently restricted to cultured species, which represents a problem for microbiome research in general though [70]. However, recent initiatives, such as BacDive [71] and BugBase [72], will help collect and standardize information on bacterial characteristics, which is needed to further generalize and apply our bias-correction approach to environmental microbiome samples. Although we could show a substantial impact of the bias correction approach in our skin microbiome samples, those samples were originally included only to test the feasibility of different extraction protocols in skin microbiome research. A more suitable validation study should include technically appropriate aliquots to show an improvement in environmental microbiome samples, as could be done, e.g., in high-input stool microbiome samples.

Extraction bias will remain a major confounder for 16S rRNA gene sequencing experiments and will not be alleviated by recent advances in sequencing technologies, such as long-read sequencing or shotgun metagenomics. To date, extraction bias is only addressed by consistently using the same lab protocol to keep the bias equally constant [2]. As an alternative approach, we propose the computational correction of extraction bias using standardized controls. We therefore promote adding at least one cell-based and a corresponding DNA-based mock community as a positive control to every sequencing experiment [30], preferably including at least three replicates to control for random events. This mock positive control should be standardized and contain enough taxa to cover diverse microbial properties, specifically covering different cell-wall properties and cell shapes and sizes, as suggested by our analysis. Ideally, additional microbial characteristics potentially relevant to extraction bias should be covered, including but not limited to endospore and capsule formation, oxygen requirements, and motility [73, 74]. Upon validation of our approach, these mocks may be used to determine protocol-specific extraction bias by bacterial properties, which is then used to correct the relative abundances of microbes present in environmental samples processed along with the mock communities, as done in the skin microbiome samples included in our study. Thus, researchers would be able to correct extraction bias internally, independent of the chosen protocol, and overcome one of the major confounders in microbiome analyses. Our findings pave the road for cross-protocol meta-analyses and for discovering more robust clinical microbiome associations.

## Supplementary Information

Supplementary Figure S1. Sequence errors are mostly assigned to *E. coli*, *L. fermentum*, or *S. enterica*, independent of the extraction protocol. Sequence errors were classified as ASVs with Levenshtein (LV) distance ≥ 1 and ≤ 8 to expected mock sequences. Point area indicates ASV relative abundance per sample. D: DNA mock sample.
Supplementary Figure S2. Chimeras are predominantly formed in high-input cell samples, independent of the extraction protocol. Bimera and trimera taxon combinations are only shown for samples with 10^8^ or 10^6^ input cells of the even and staggered mock community. Chimeras were defined as ASVs with Levenshtein (LV) distance ≥ 8, and > 95% sequence identity with at least two expected mock taxa. Point area indicates each chimera combination’s relative abundance per sample. D: DNA mock sample.
Supplementary Figure S3. Sequence distances between mock and spike-in expected sequences. Levenshtein (LV) distance specifies the number of substitutions or indels between sequences, with zero indicating identical sequences. Clustering analysis of mean LV distances highlights closely related expected sequences between *E. coli*, *S. enterica*, and *P. aeruginosa*, and between *S. aureus*, *B. subtilis*, and *L. monocytogenes*. Values represent mean LV distances between 16S rRNA copy variants of two species, based on reference sequences provided by ZymoResearch and cut to 279 bp of the V1-V3 region. Values in the diagonal indicate mean LV distances between copy variants within each species. Clustering was performed with Euclidean distance and complete linkage.
Supplementary Figure S4. Most contaminant reads in mock samples and negative controls originate from cross-contamination of skin samples or from extraction buffers. Contaminating ASVs were clustered into four groups by kmeans. Clusters were assigned to buffer origin by their distinct and consistent appearance across samples of the same extraction buffer, and to skin origin by their high relative abundance in skin microbiome samples (indicated by darker color). Shown are ASVs previously categorized as ‘Unclassified’, i.e., with LV distance ≥ 8 and ≤ 95% identity with expected mock taxa sequences, and present in at least three non-skin samples (mock or negative controls). Point area indicates ASV relative abundance per sample.
Supplementary Figure S5. Sample composition in the staggered or spike-in mock community is significantly affected by extraction protocol, kit, or lysis condition, but microbiome compositions between two subjects are even more distinct than between extraction protocols. In the staggered mock community (A-E), beta diversity analysis revealed significant differences in global mock composition between lysis conditions (D), but not between dilutions (A), protocols (B), kits (C), or buffers (E). In the spike-in community (F-J), protocols (G) and kits (H) significantly affected sample compositions, but not dilutions (F), lysis conditions (I), or buffers (J). In contrast, in the skin microbiome samples (K–O), significant differences in global microbiome composition were only detected between the two subjects (K), but not between any of the extraction protocol variables (L-O). Beta diversity was performed only on mock taxa with LV ≤ 4 to any expected mock sequence, and on genus level of the DADA2-based taxonomic annotation in the skin samples, and is visualized by PCoA on Bray–Curtis dissimilarities. Polygonal shaded areas connect samples of the same group, and p-values are derived from PERMANOVA tests with 500 permutations. Q: Qiagen extraction kit, Z: ZymoResearch extraction kit, S: ‘soft’ lysis condition, T: ‘tough’ lysis condition, q: Qiagen/Stratec buffer, z: ZymoResearch buffer.
Supplementary Figure S6. The magnitude of extraction bias varies between extraction protocols, but also between mock communities. Extraction bias, measured as Bray–Curtis distance to the DNA mock composition, varies between extraction protocols in the even (A) and staggered (B) mock community, with no protocol achieving a perfect representation (no bias). Substantial differences in protocol bias were observed between the two mock communities, independent of the chosen distance measure (C). Boxes (A, B) denote the median and interquartile range (IQR), whiskers represent values up to 1.5 times the IQR, and dots indicate individual samples. A darker red background color (C) indicates higher extraction bias per protocol.
Supplementary Figure S7. Distance to the DNA or expected mock composition is reduced after applying morphology-based correction of extraction bias in all mock communities, shown for all protocols. Extraction bias per protocol was calculated from the 10 6 even mock samples, summarized by bacterial morphology group, and applied to the 10^6^ even mock sample (internal correction, A), but also to different samples of the same mock (even mock 10^8^, 10^5^, 10^4^ cells, B), different samples of a different mock (staggered mock, C), and to different samples with different taxa (spike-in mock, D). Extraction bias was measured by Bray–Curtis dissimilarity to the DNA mock composition (even mock, staggered mock) or to the expected mock composition (spike-in mock). Boxes denote the median and interquartile range (IQR), whiskers represent values up to 1.5 times the IQR, dots indicate individual samples.
Supplementary Figure S8. Impact of the bacterial morphology-based correction of extraction bias on skin microbiome samples. Both the overall sample compositions on genus level (A) and the relative abundance of the top three skin microbiome taxa on species level (B) changed considerably after correcting for extraction bias. Sample composition (A) is shown for the top 10 skin genera, with the remaining genera summarized as Others. Q: Qiagen extraction kit, Z: ZymoResearch extraction kit, S: ‘soft’ lysis condition, T: ‘tough’ lysis condition, q: Qiagen/Stratec buffer, z: ZymoResearch buffer.
Supplementary Table S1. Correction factors of extraction bias per protocol and taxon, based on bacterial morphology group. Darker pink background color indicates more severely underrepresented taxa that are corrected up, and darker blue background color indicates more severely overrepresented taxa that are corrected down, in their relative abundance by multiplying with the factor presented in the table. Q: Qiagen extraction kit, Z: ZymoResearch extraction kit, S: ‘soft’ lysis condition, T: ‘tough’ lysis condition, q: Qiagen/Stratec buffer, z: ZymoResearch buffer.

## Data Availability

The raw sequencing data used in this manuscript has been deposited at the European Nucleotide Archive (ENA) under accession number PRJEB67827 (https://www.ebi.ac.uk/ena/browser/view/PRJEB67827) and are additionally available under https://osf.io/ykrbp/, hosted at the Open Science Framework platform (OSF). Data analysis scripts used in this manuscript are available on Github (https://github.com/LuiseRauer/Extraction-bias-correction).
