# Supplementary material for: De-biasing microbiome sequencing data: bacterial morphology-based correction of extraction bias and correlates of chimera formation
Source: Microbiome. 2025 Feb 4;13:38. doi: 10.1186/s40168-024-01998-4 (PMC11792448; doi:10.1186/s40168-024-01998-4)
Supplement: Supplementary file 2 — Supplementary Figure S1. Sequence errors are mostly assigned to E. coli, L. fermentum, or S. enterica, independent of the extraction protocol. Sequence errors were classified as ASVs with Levenshtein (LV) distance ≥ 1 and ≤ 8 to expected mock sequences. Point area indicates ASV relative abundance per sample. D: DNA mock sample. Supplementary Figure S2. Chimeras are predominantly formed in high-input cell samples, independent of the extraction protocol. Bimera and trimera taxon combinations are only shown for samples with 108 or 106 input cells of the even and staggered mock community. Chimeras were defined as ASVs with Levenshtein (LV) distance ≥ 8, and > 95% sequence identity with at least two expected mock taxa. Point area indicates each chimera combination’s relative abundance per sample. D: DNA mock sample. Supplementary Figure S3. Sequence distances between mock and spike-in expected sequences. Levenshtein (LV) distance specifies the number of substitutions or indels between sequences, with zero indicating identical sequences. Clustering analysis of mean LV distances highlights closely related expected sequences between E. coli, S. enterica, and P. aeruginosa, and between S. aureus, B. subtilis, and L. monocytogenes. Values represent mean LV distances between 16S rRNA copy variants of two species, based on reference sequences provided by ZymoResearch and cut to 279 bp of the V1-V3 region. Values in the diagonal indicate mean LV distances between copy variants within each species. Clustering was performed with Euclidean distance and complete linkage. Supplementary Figure S4. Most contaminant reads in mock samples and negative controls originate from cross-contamination of skin samples or from extraction buffers. Contaminating ASVs were clustered into four groups by kmeans. Clusters were assigned to buffer origin by their distinct and consistent appearance across samples of the same extraction buffer, and to skin origin by their high relative abundance in skin mi [file 40168_2024_1998_MOESM1_ESM.docx]

**
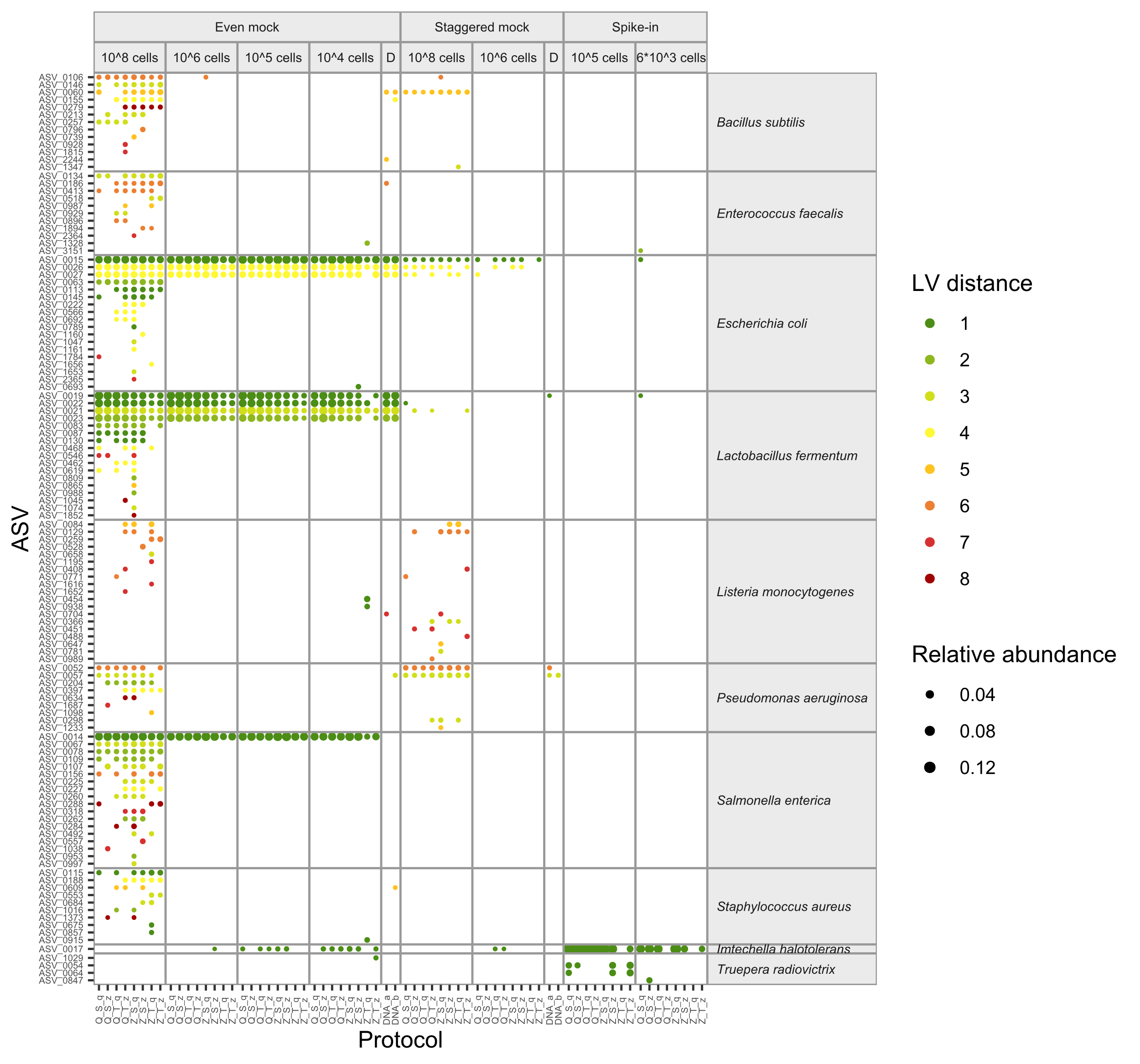
**

**Supplementary Figure S1. Sequence errors are mostly assigned to *E. coli*, *L. fermentum*, or *S. enterica****,* **independent of the extraction protocol.** Sequence errors were classified as ASVs with Levenshtein (LV) distance ≥1 and ≤ 8 to expected mock sequences. Point area indicates ASV relative abundance per sample. D: DNA mock sample.

**
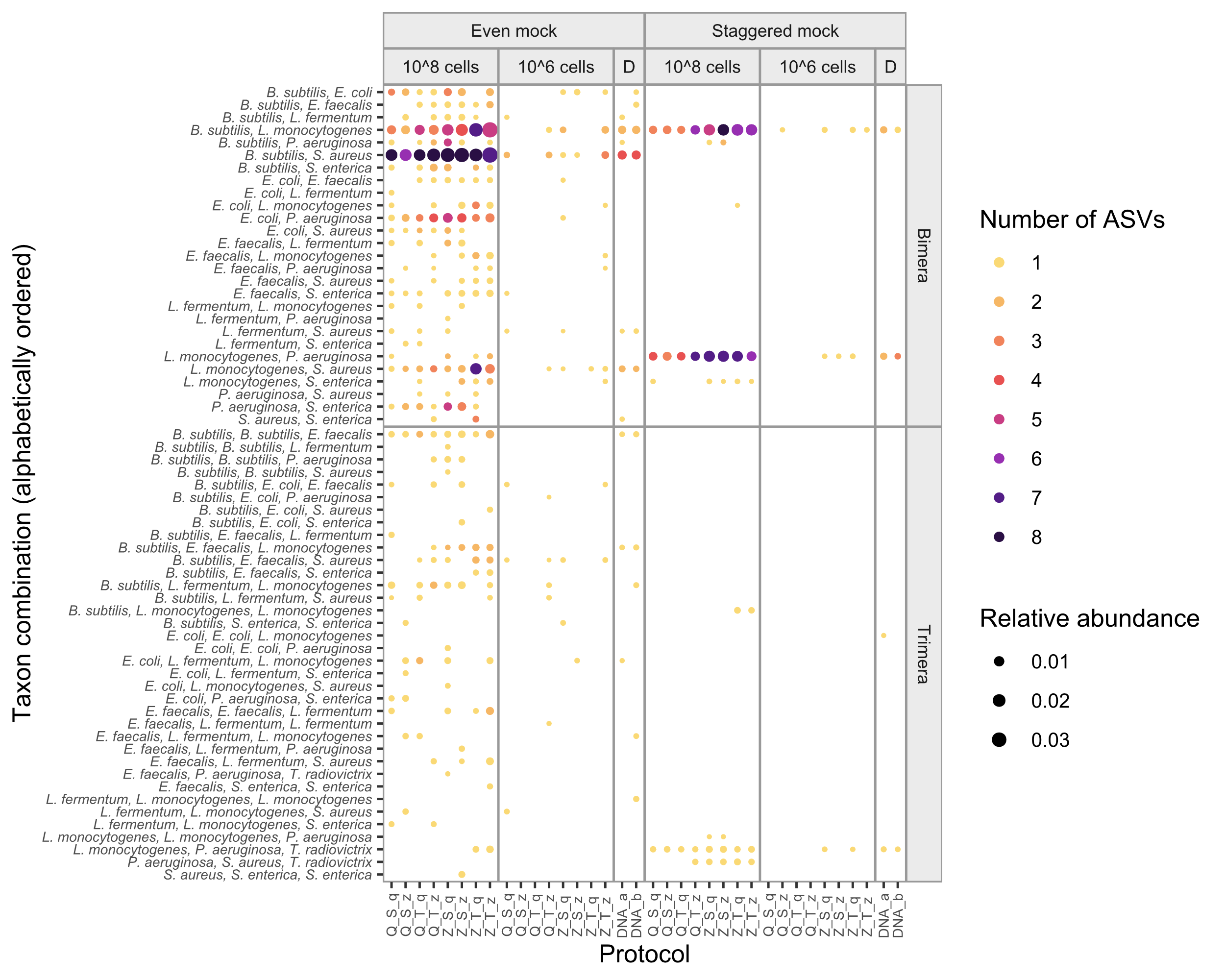
**

**Supplementary Figure S2. Chimeras are predominantly formed in high-input cell samples, independent of the extraction protocol.** Bimera and trimera taxon combinations are only shown for samples with 10^8^ or 10^6^ input cells of the even and staggered mock community. Chimeras were defined as ASVs with Levenshtein (LV) distance ≥ 8, and > 95% sequence identity with at least two expected mock taxa. Point area indicates each chimera combination’s relative abundance per sample. D: DNA mock sample.

**
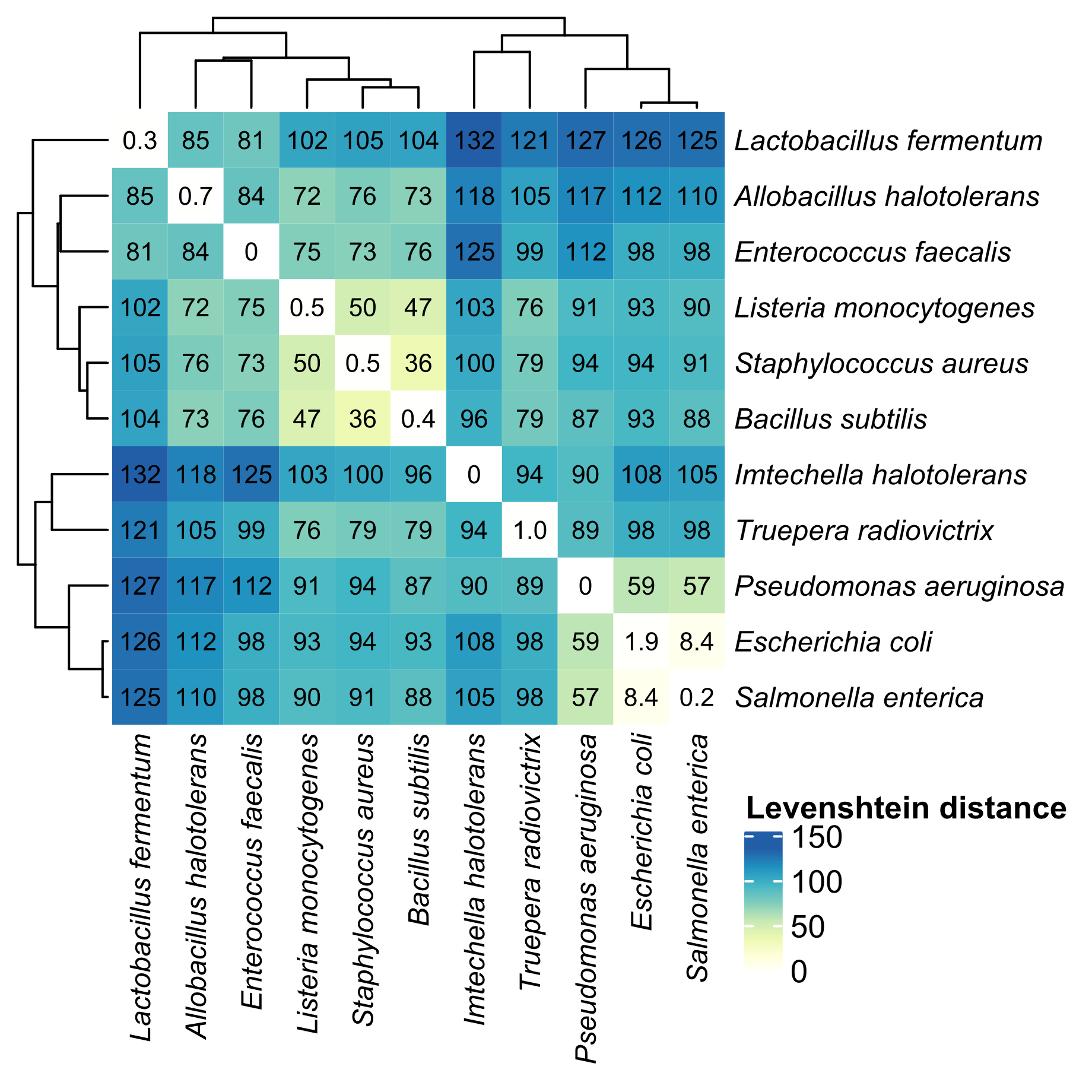
**

**Supplementary Figure S3. Sequence distances between mock and spike-in expected sequences**. Levenshtein (LV) distance specifies the number of substitutions or indels between sequences, with zero indicating identical sequences. Clustering analysis of mean LV distances highlights closely related expected sequences between *E. coli*, *S. enterica*, and *P. aeruginosa*, and between *S. aureus*, *B. subtilis*, and *L. monocytogenes*. Values represent mean LV distances between 16S rRNA copy variants of two species, based on reference sequences provided by ZymoResearch and cut to 279 bp of the V1-V3 region. Values in the diagonal indicate mean LV distances between copy variants within each species. Clustering was performed with Euclidean distance and complete linkage.

**
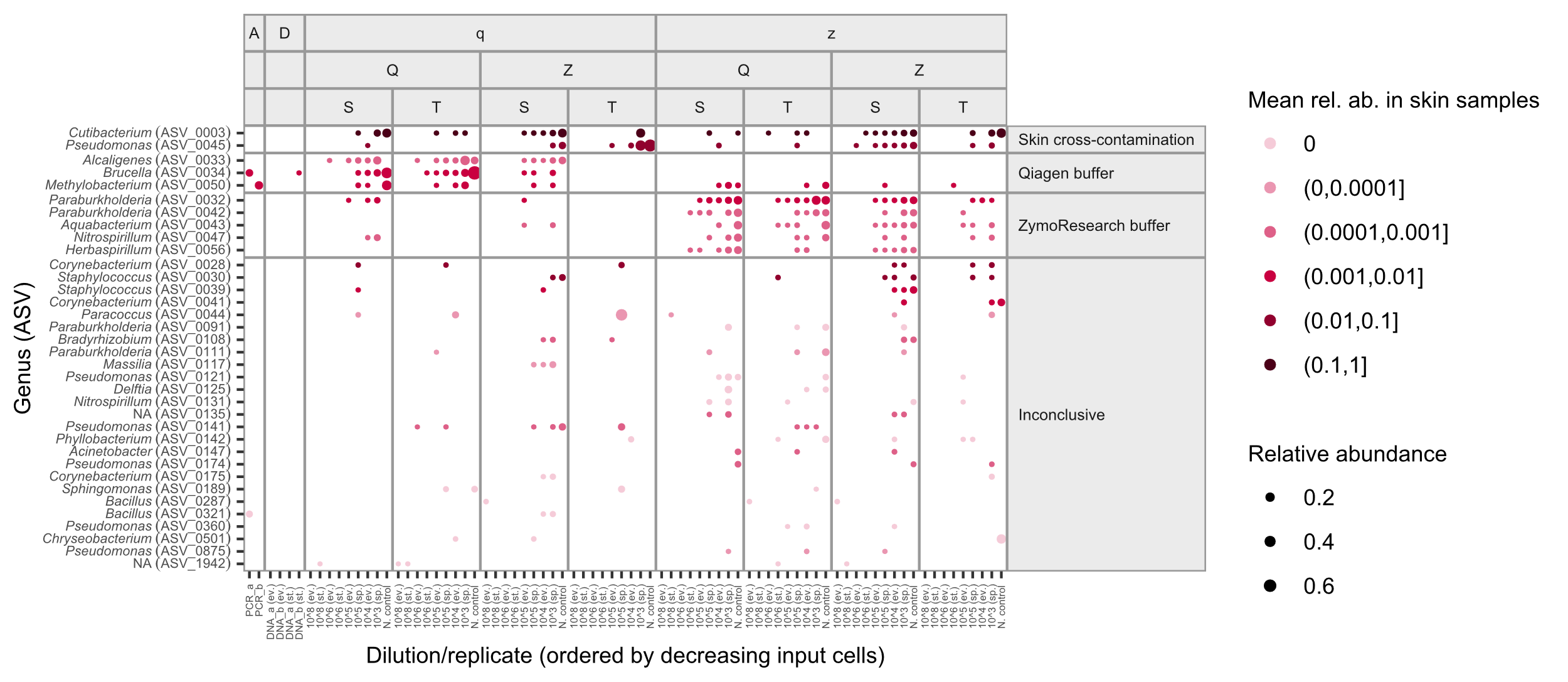
**

**Supplementary Figure S4. Most contaminant reads in mock samples and negative controls originate from cross-contamination of skin samples or from extraction buffers**. Contaminating ASVs were clustered into four groups by kmeans. Clusters were assigned to buffer origin by their distinct and consistent appearance across samples of the same extraction buffer, and to skin origin by their high relative abundance in skin microbiome samples (indicated by darker color). Shown are ASVs previously categorized as ‘Unclassified’, i.e., with LV distance ≥ 8 and ≤ 95% identity with expected mock taxa sequences, and present in at least three non-skin samples (mock or negative controls). Point area indicates ASV relative abundance per sample.

**Staggered mock**

**Spike-in**

**Skin**


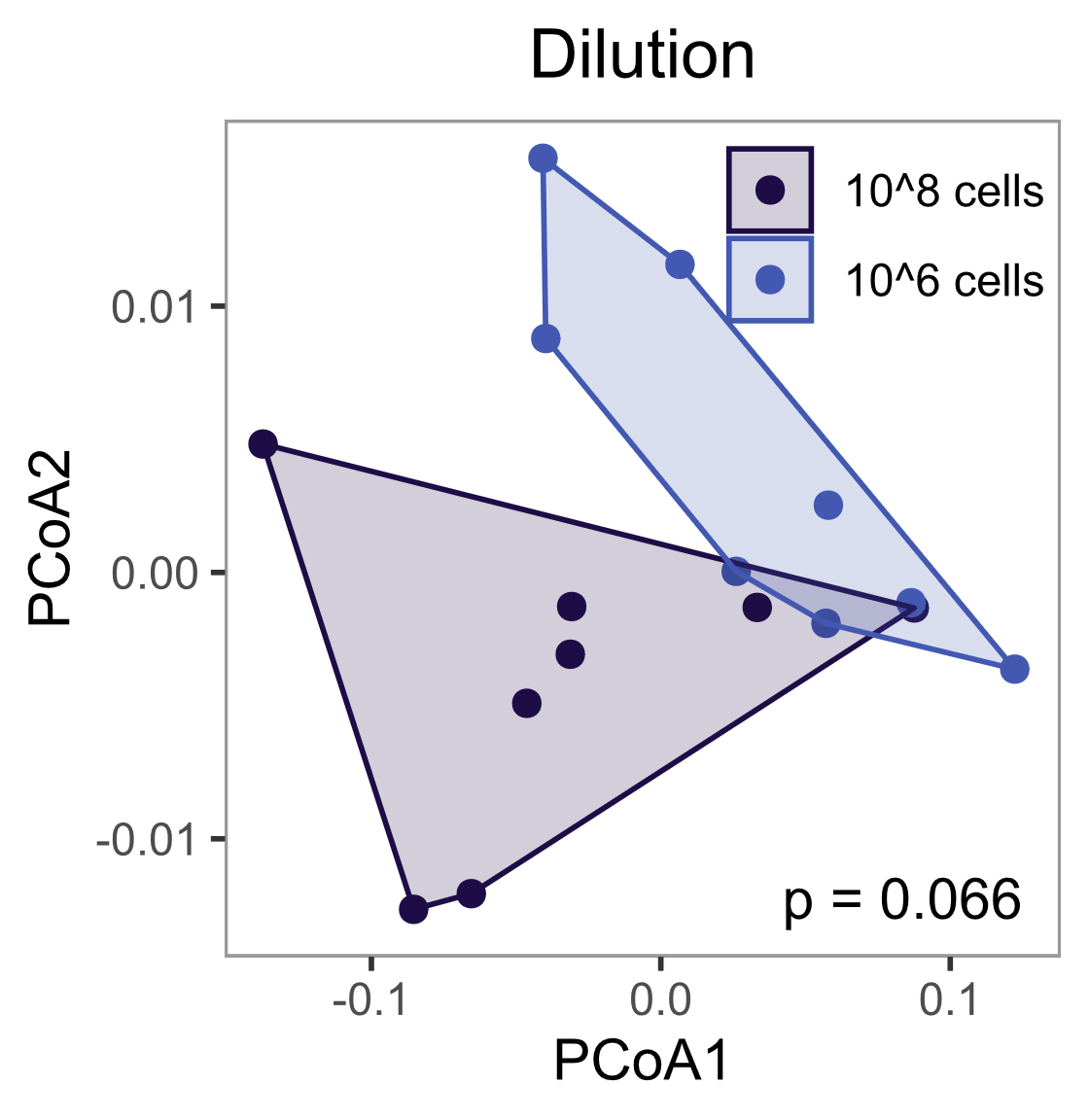

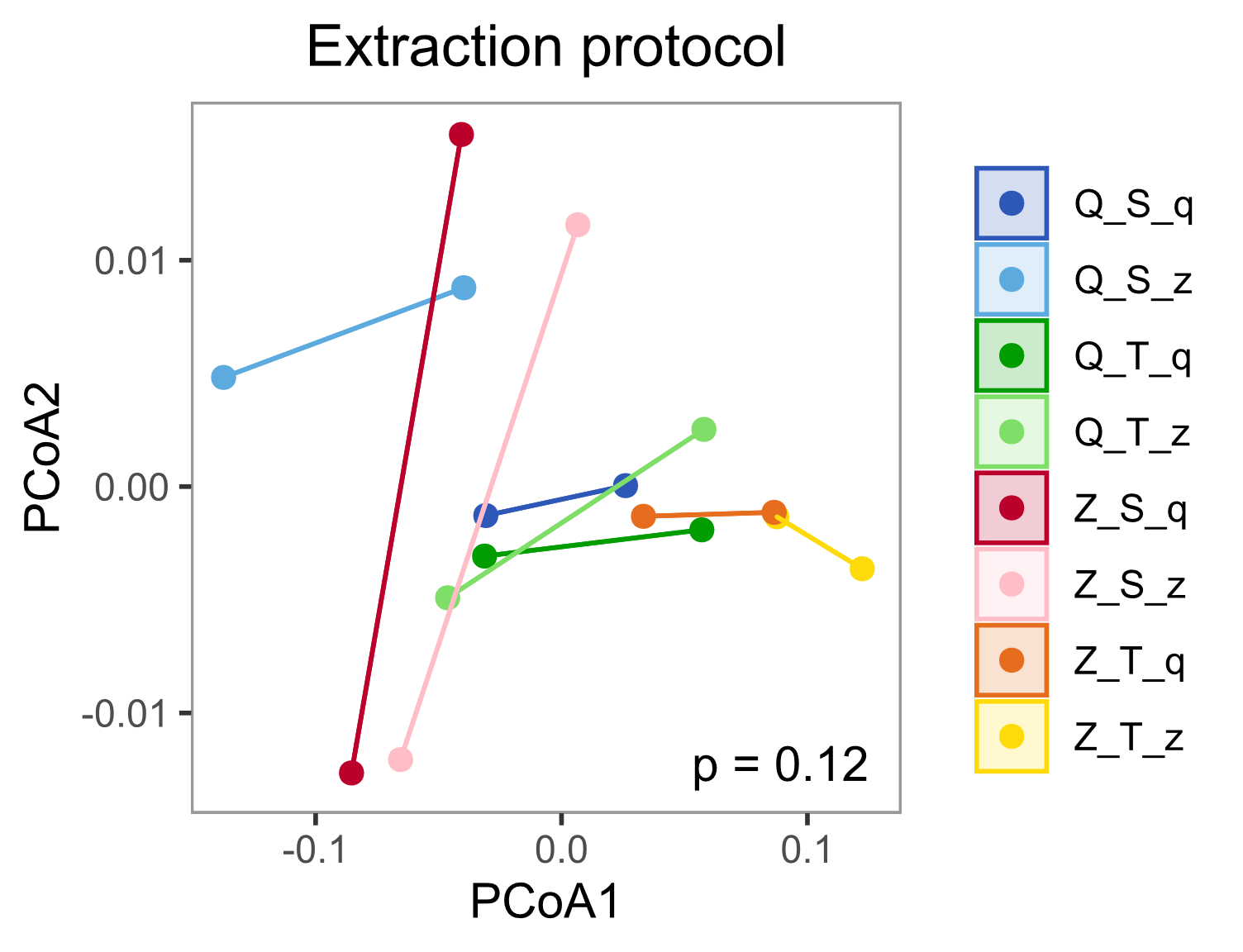

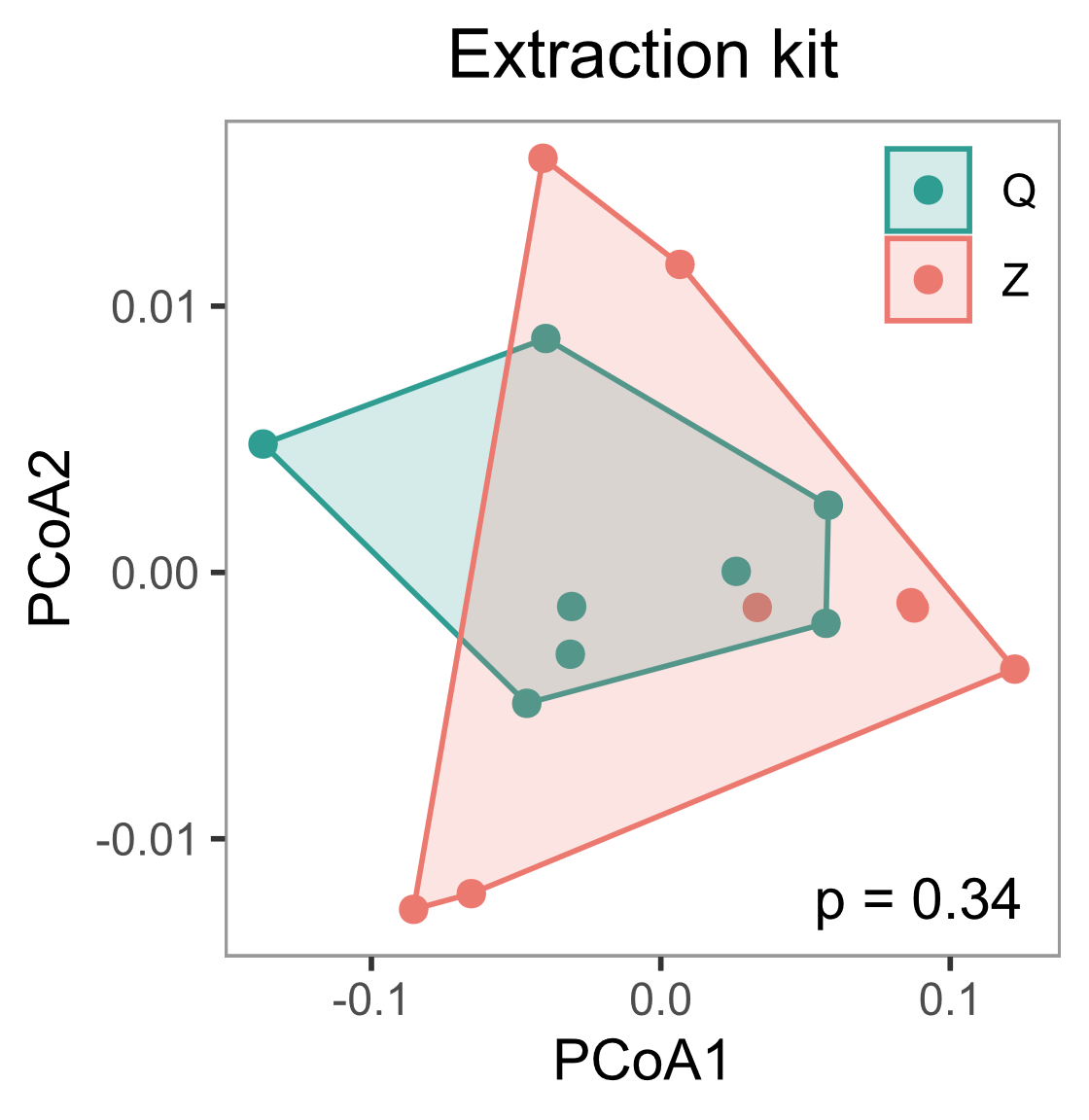

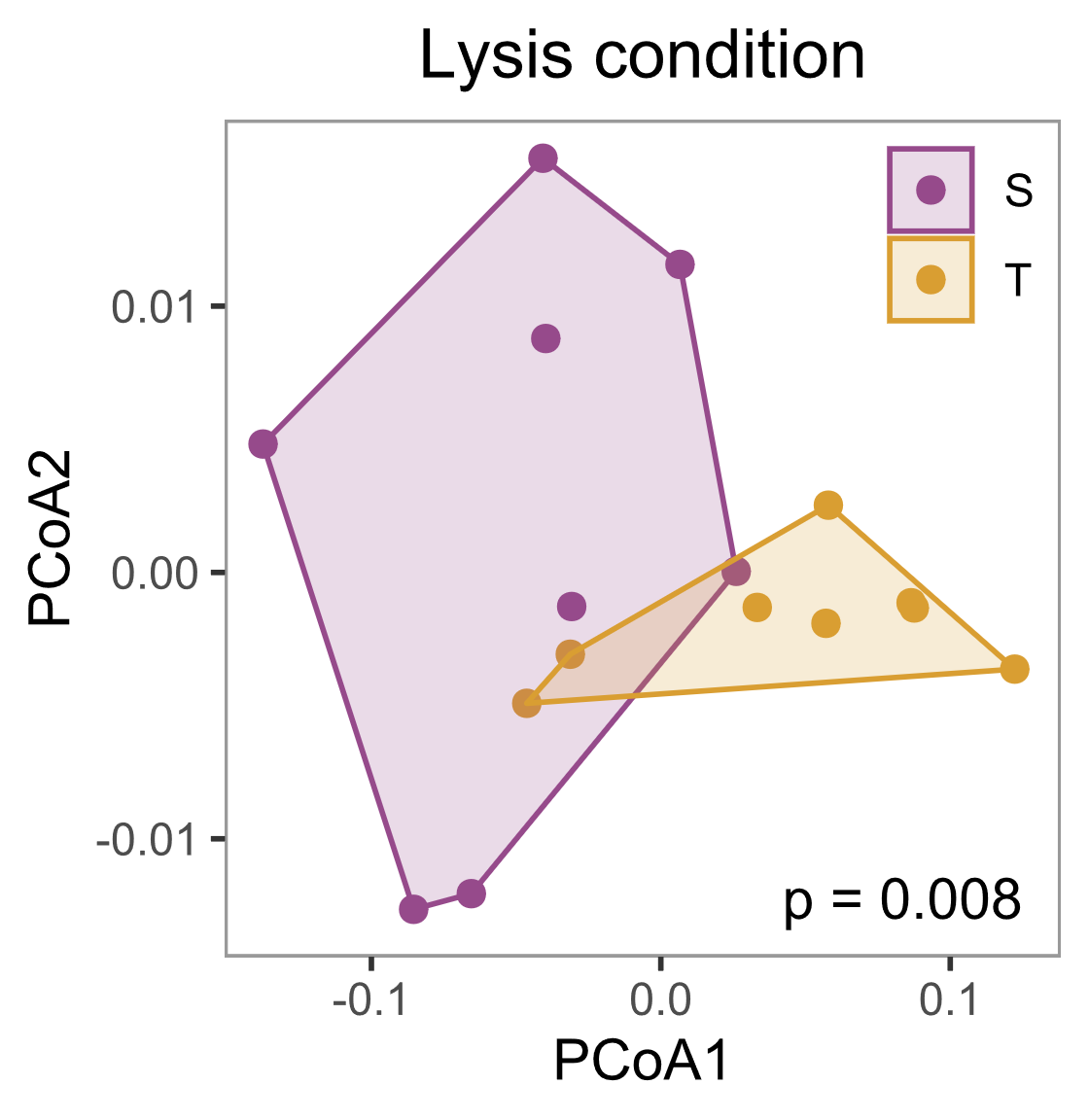

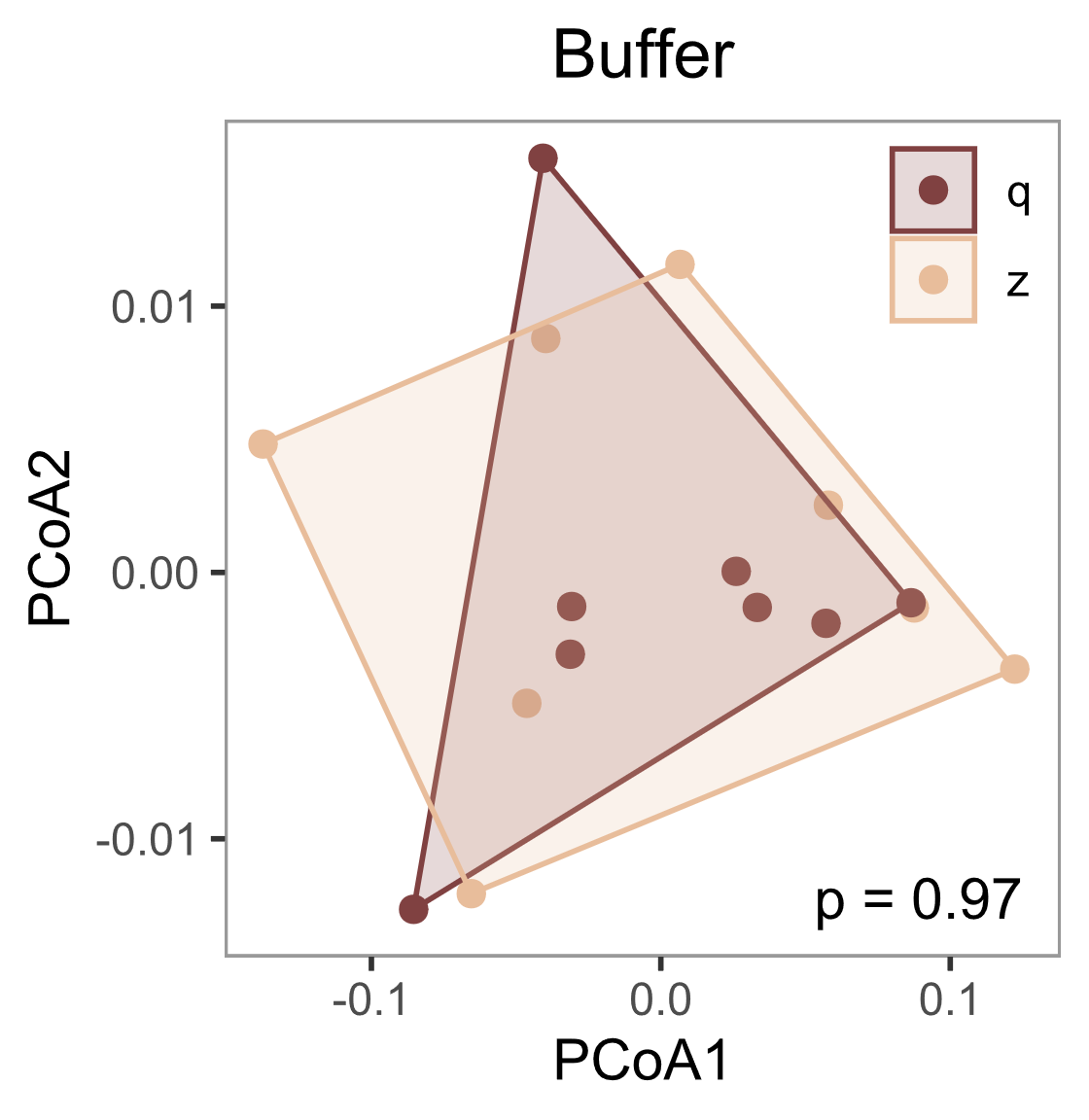

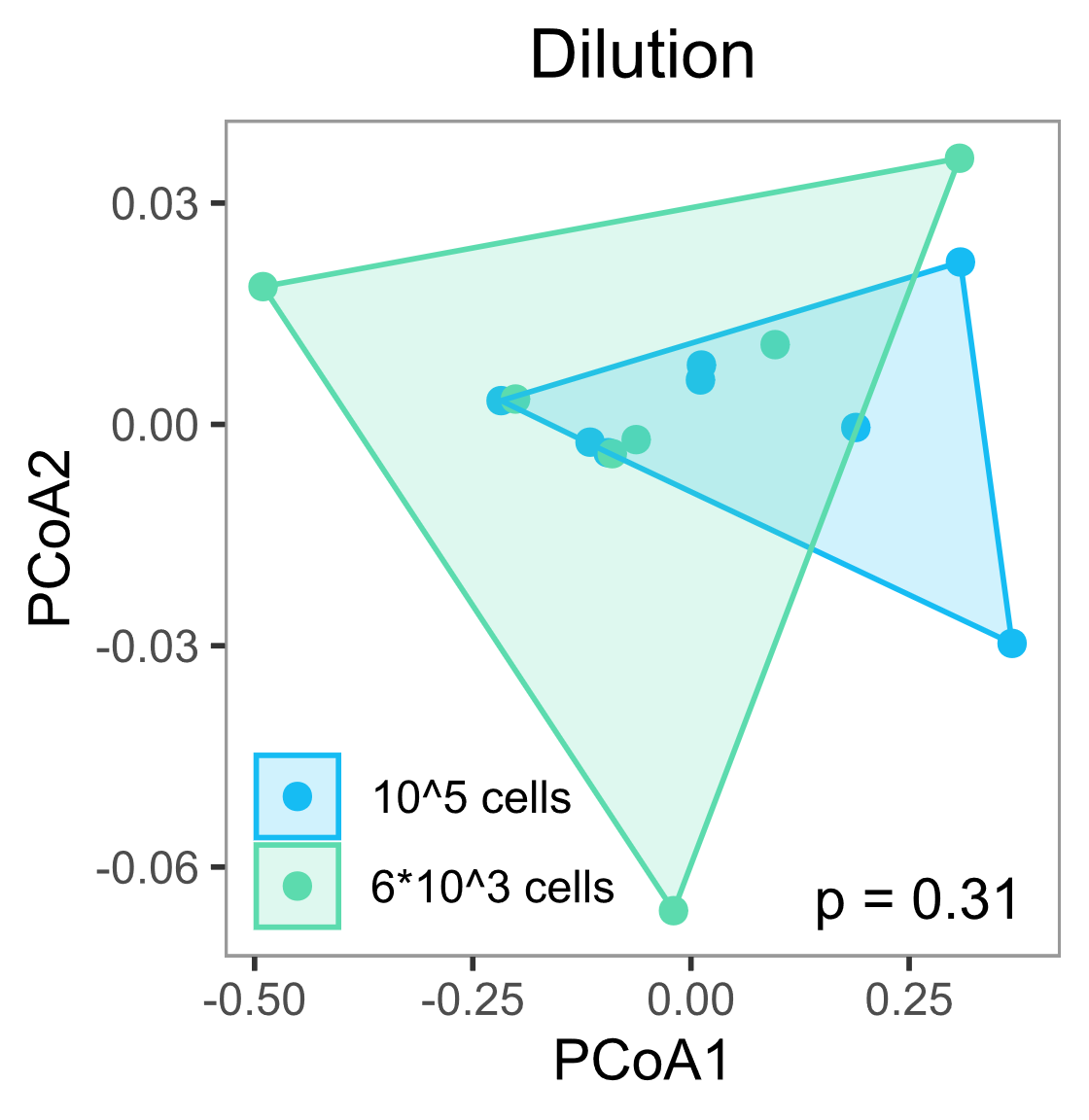

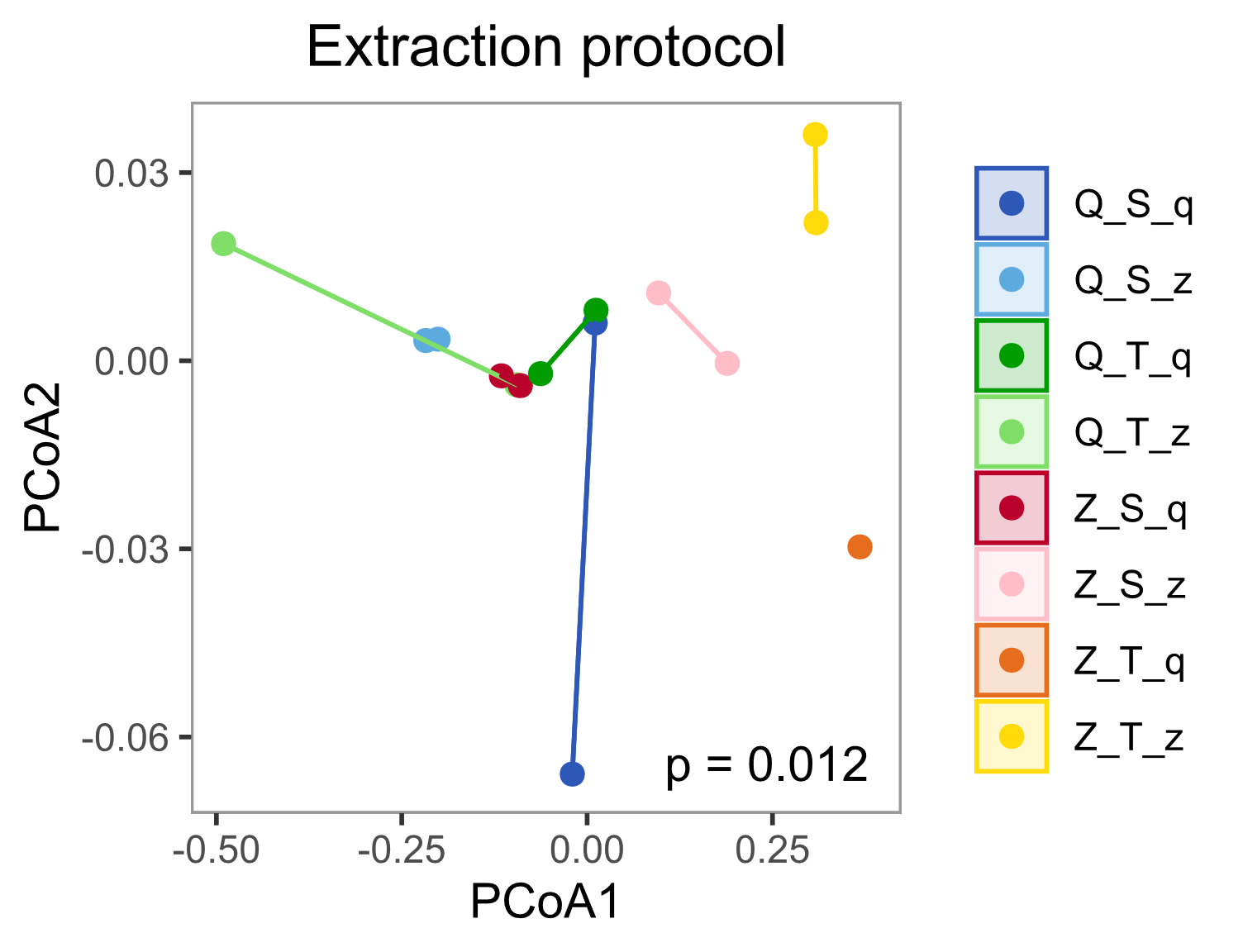

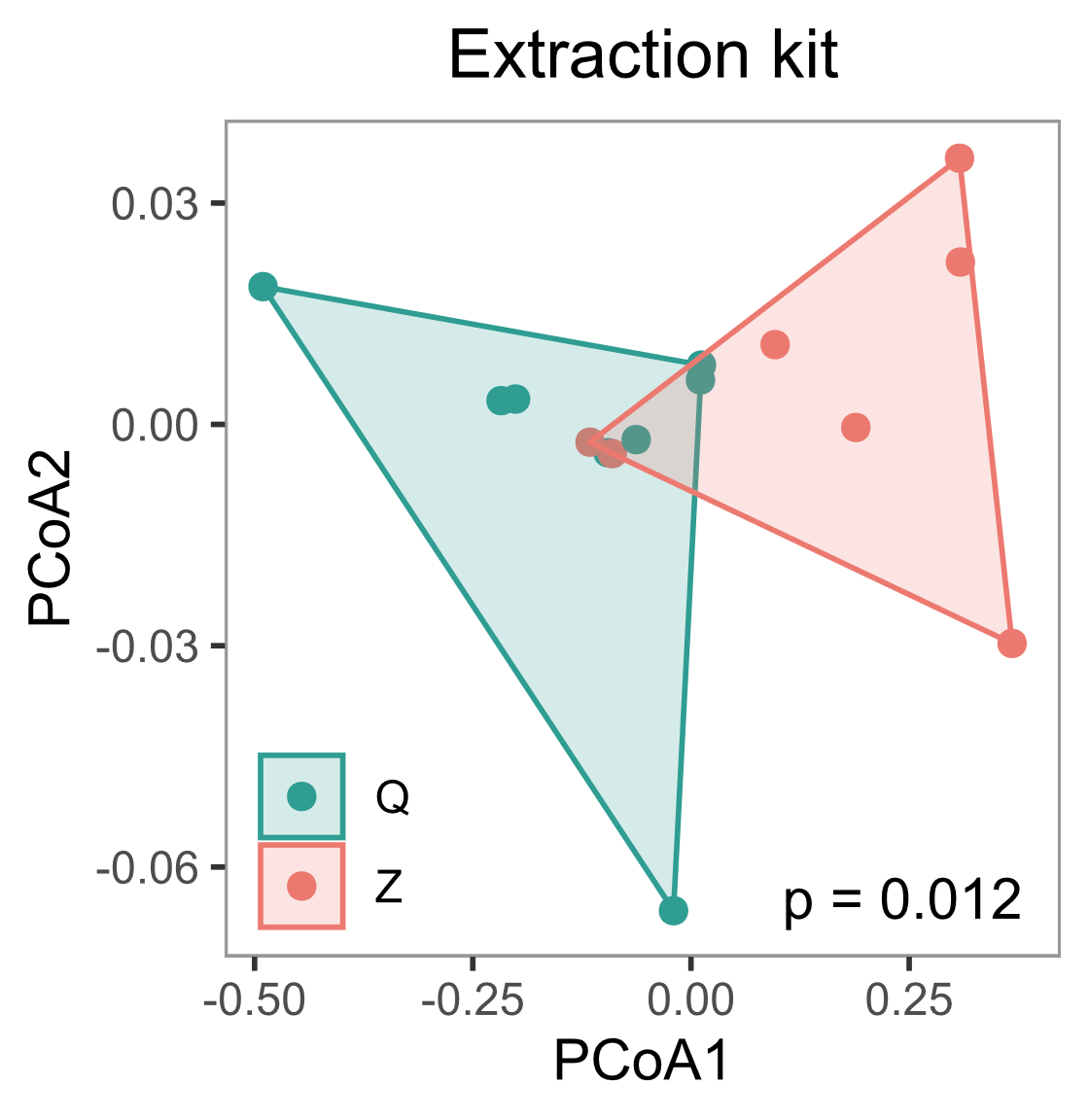

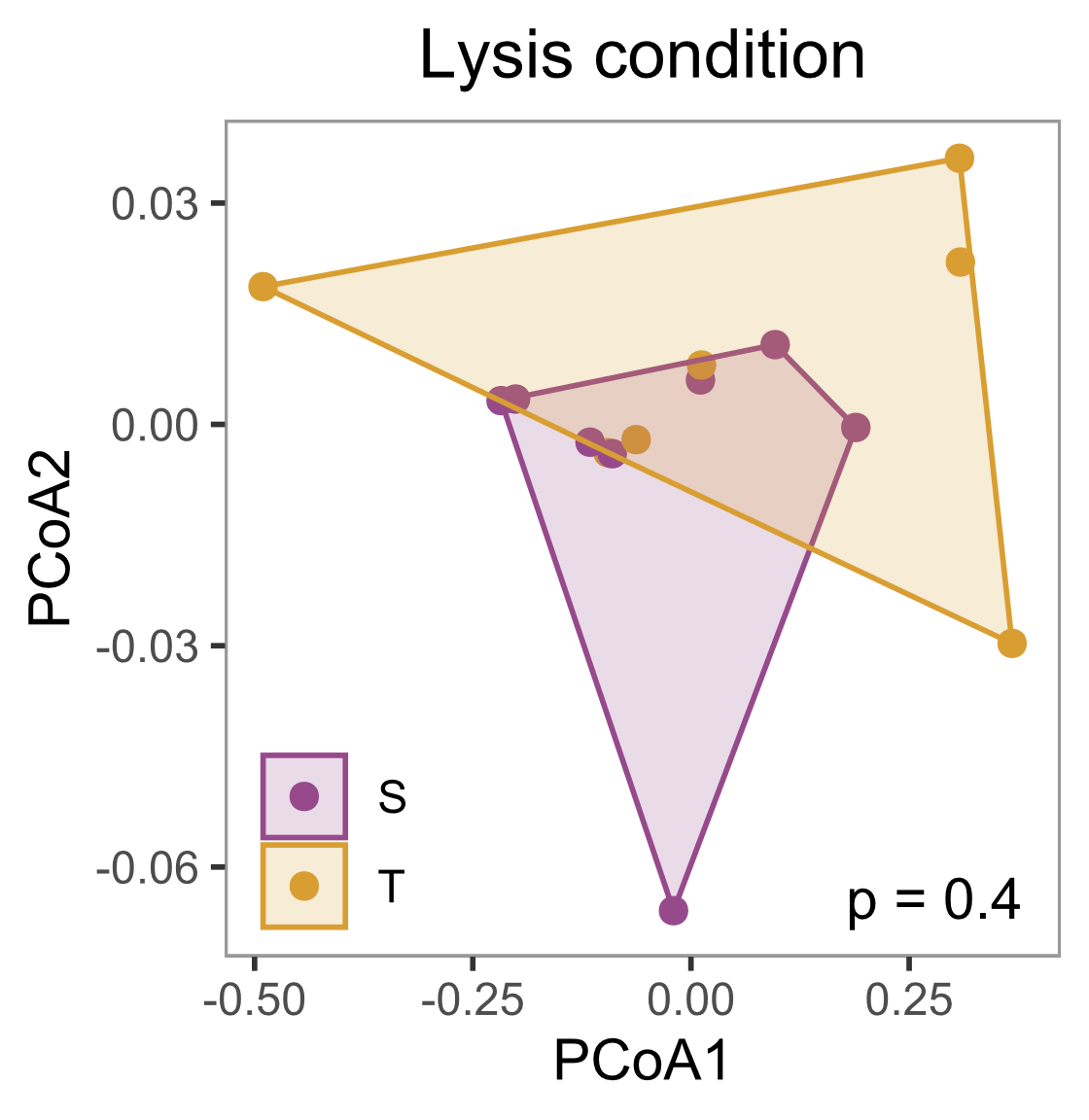

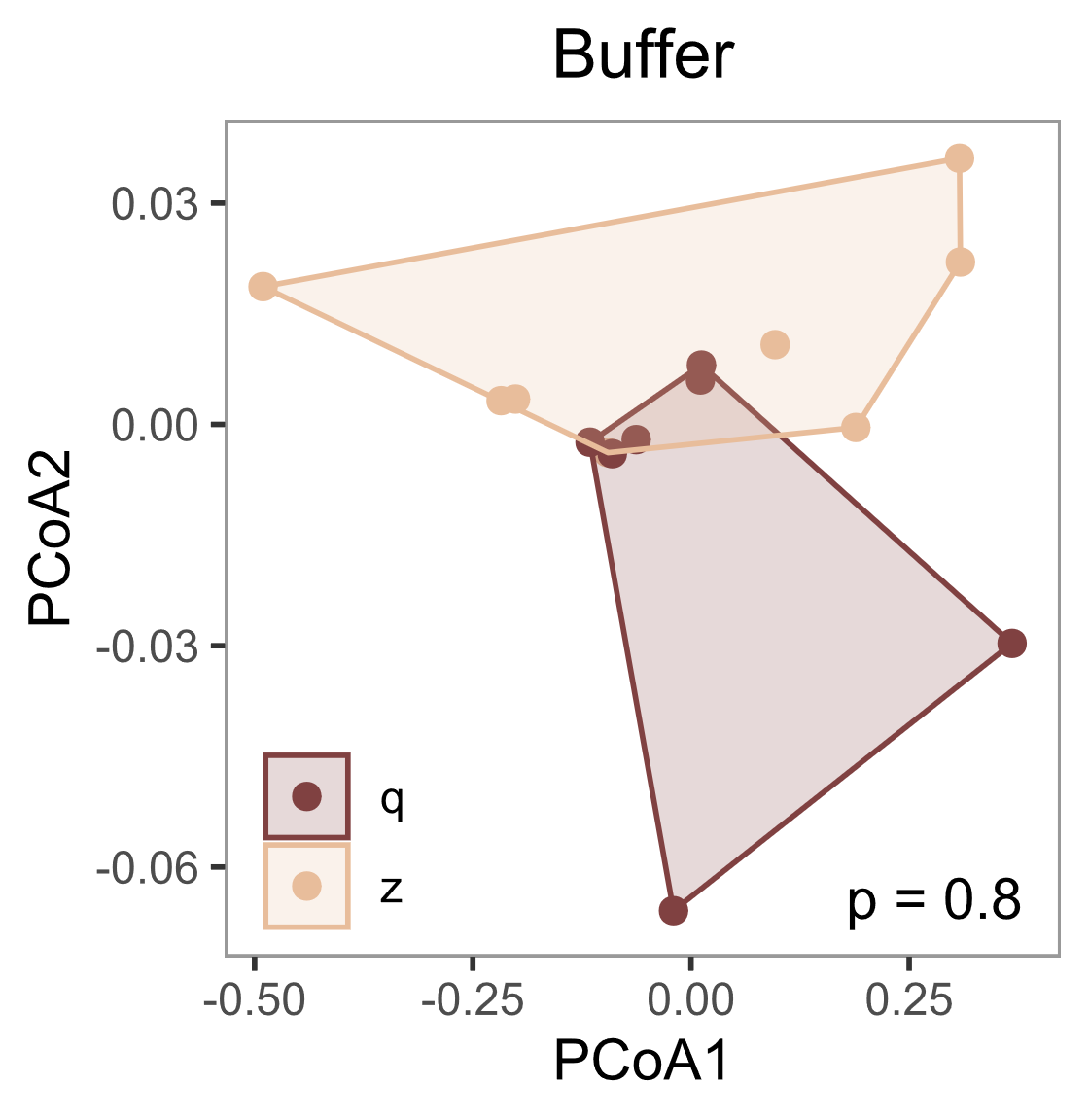

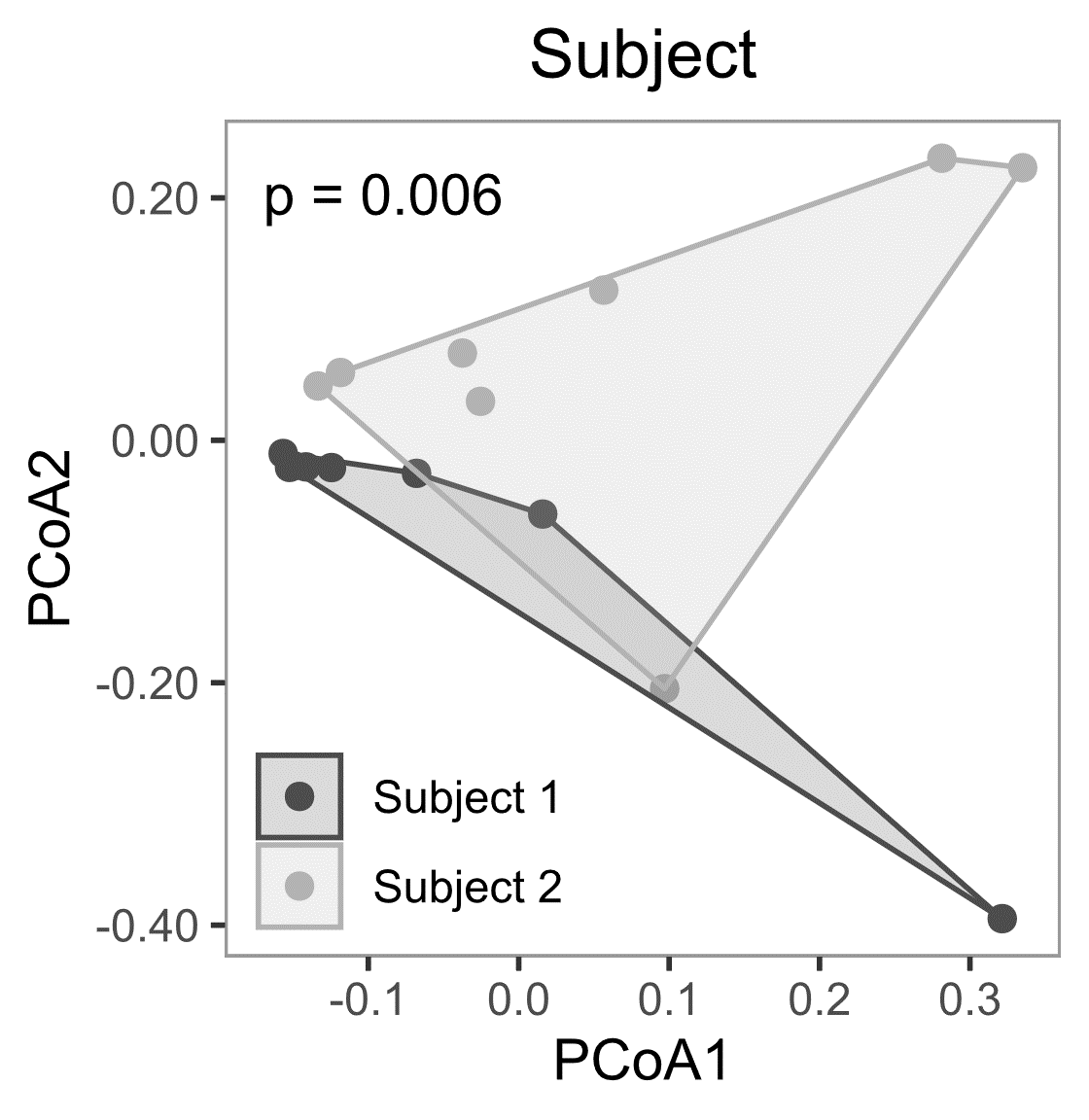

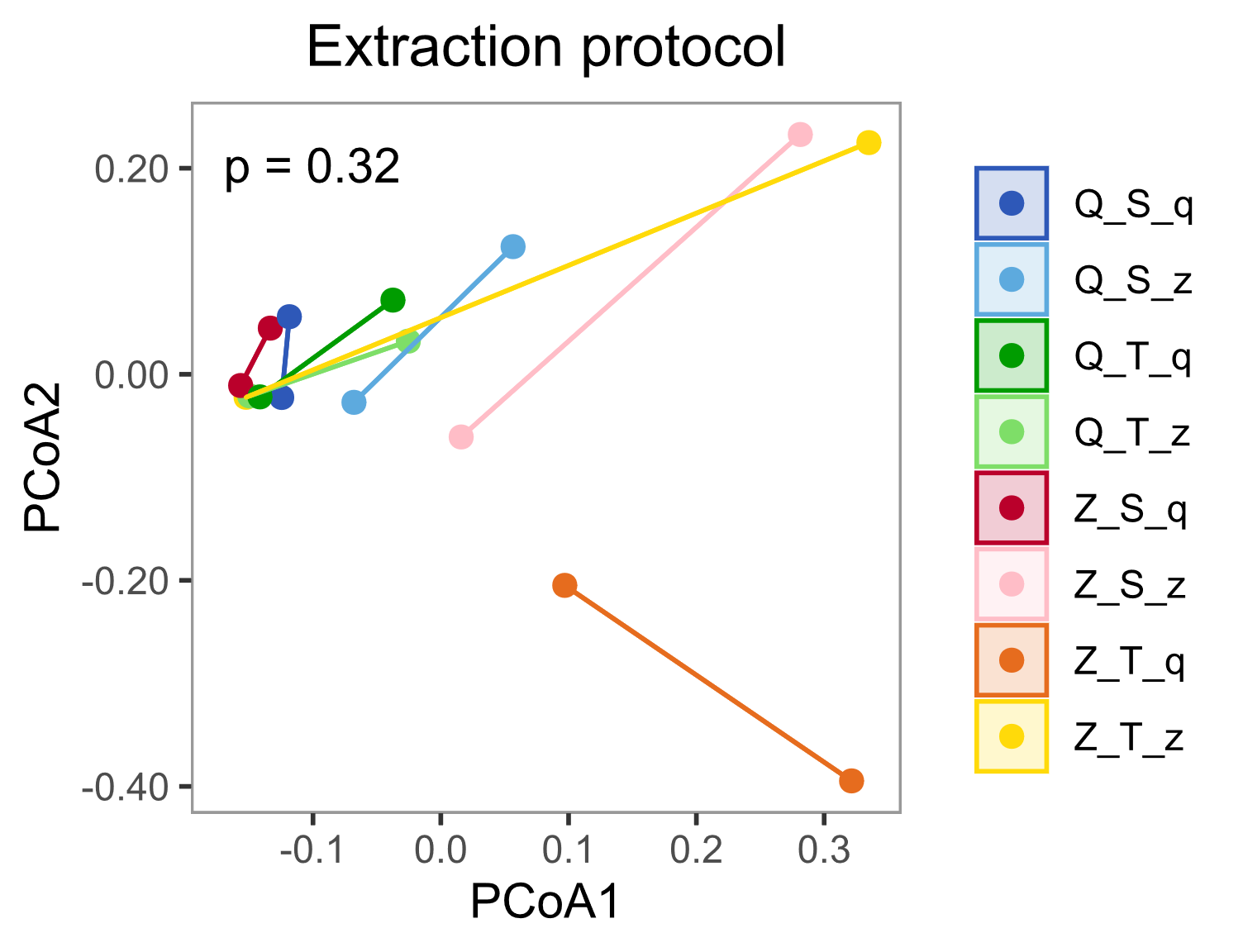

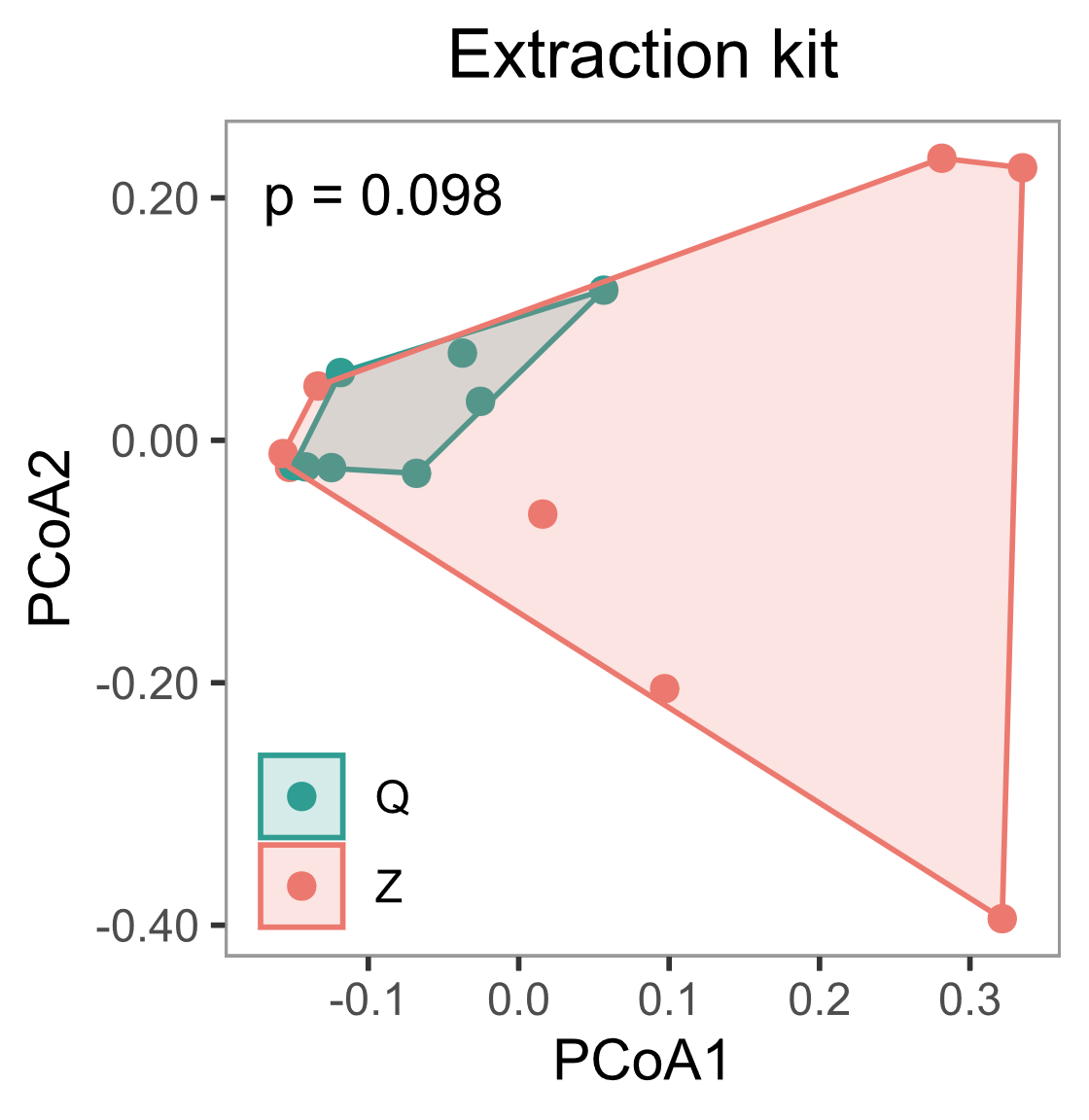

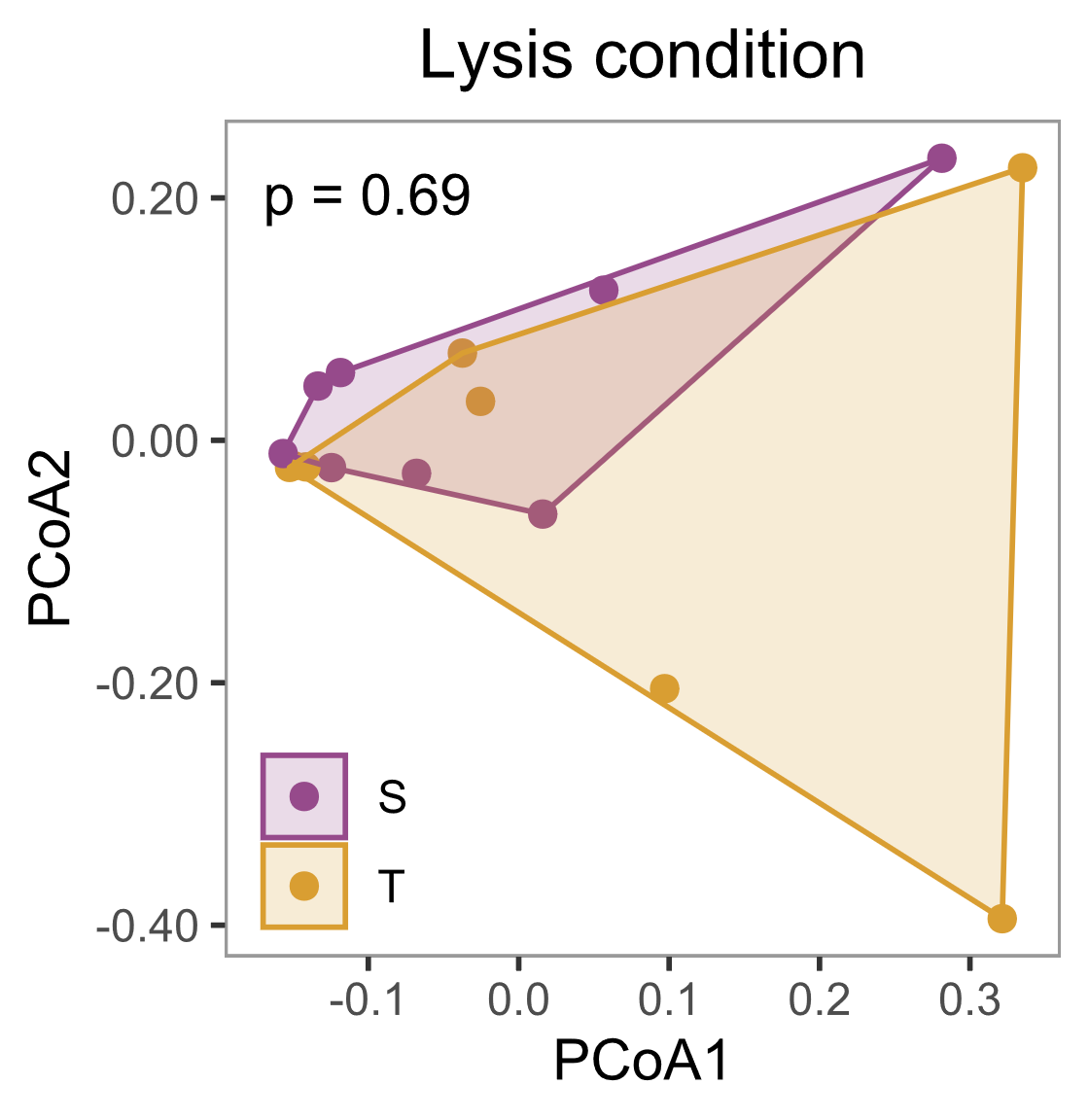

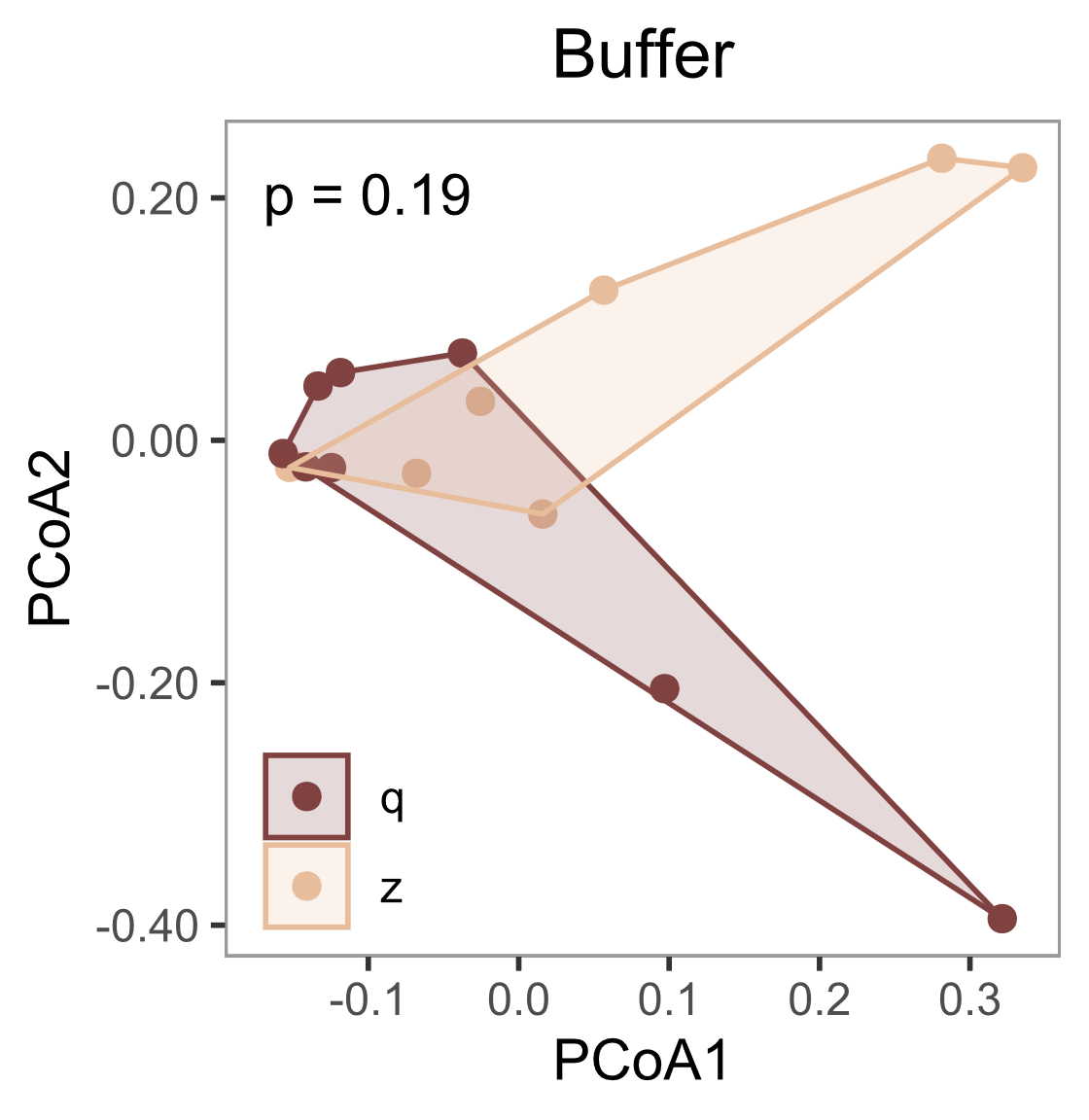


**A**

**B**

**C**

**D**

**E**

**F**

**G**

**H**

**I**

**J**

**K**

**L**

**M**

**N**

**O**

**Supplementary Figure S5.** **Sample composition in the staggered or spike-in mock community is significantly affected by extraction protocol, kit, or lysis condition, but microbiome compositions between two subjects are even more distinct than between extraction protocols.** In the staggered mock community (**A-E**), beta diversity analysis revealed significant differences in global mock composition between lysis conditions (**D**) but not between dilutions (**A**), protocols (**B**), kits (**C**), or buffers (**E**). In the spike-in community (**F-J**), protocols (**G**) and kits (**H**) significantly affected sample compositions but not dilutions (**F**), lysis conditions (**I**), or buffers (**J**). In contrast, in the skin microbiome samples (**K-O**), significant differences in global microbiome composition were only detected between the two subjects (**K**), but not between any of the extraction protocol variables (**L-O**). Beta diversity was performed only on mock taxa with LV ≤ 4 to any expected mock sequence, and on genus level of the DADA2-based taxonomic annotation in the skin samples, and is visualized by PCoA on Bray-Curtis dissimilarities. Polygonal shaded areas connect samples of the same group, and p-values are derived from PERMANOVA tests with 500 permutations. Q: Qiagen extraction kit, Z: ZymoResearch extraction kit, S: ‘soft’ lysis condition, T: ‘tough’ lysis condition, q: Qiagen/Stratec buffer, z: ZymoResearch buffer.

**
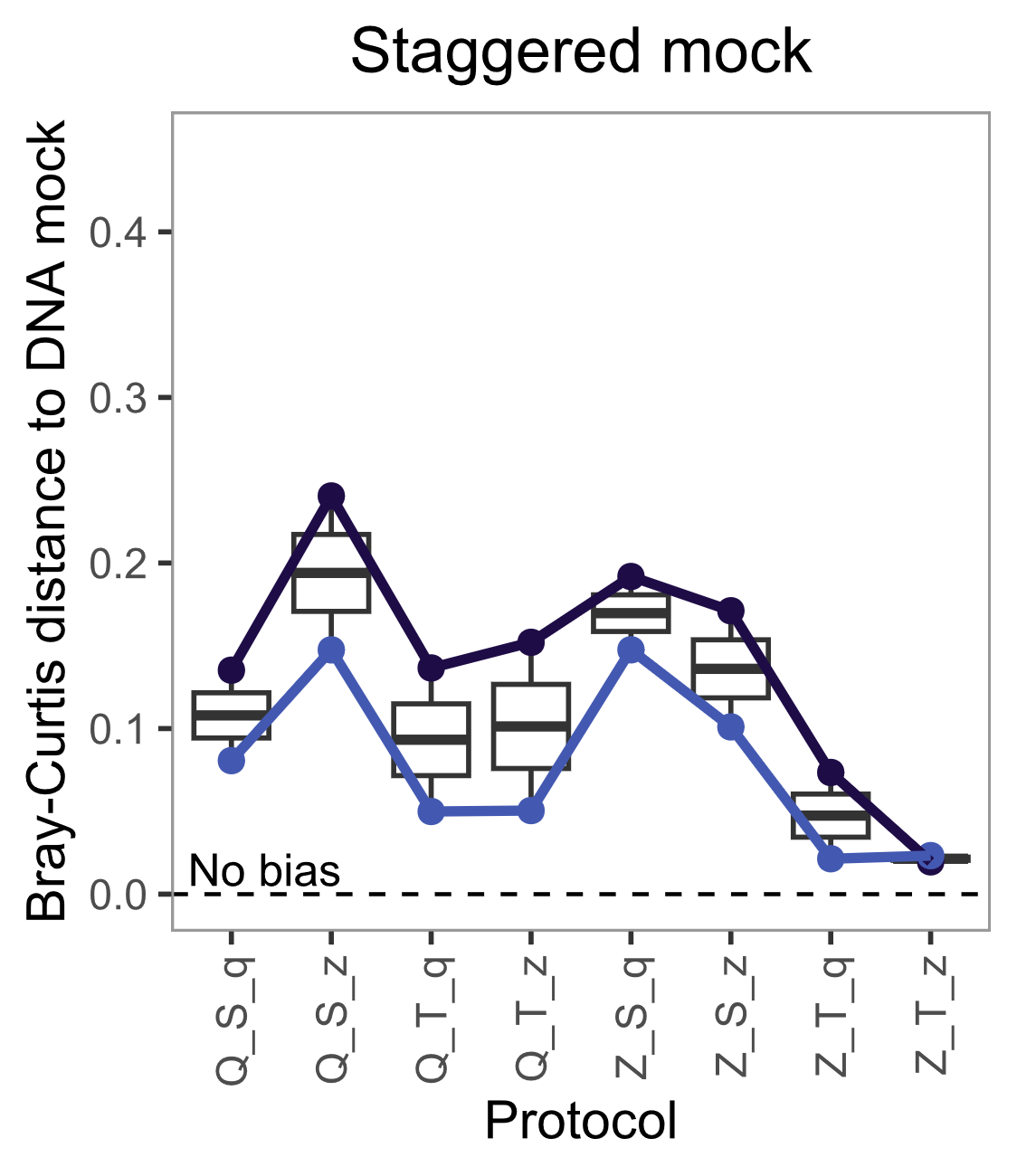

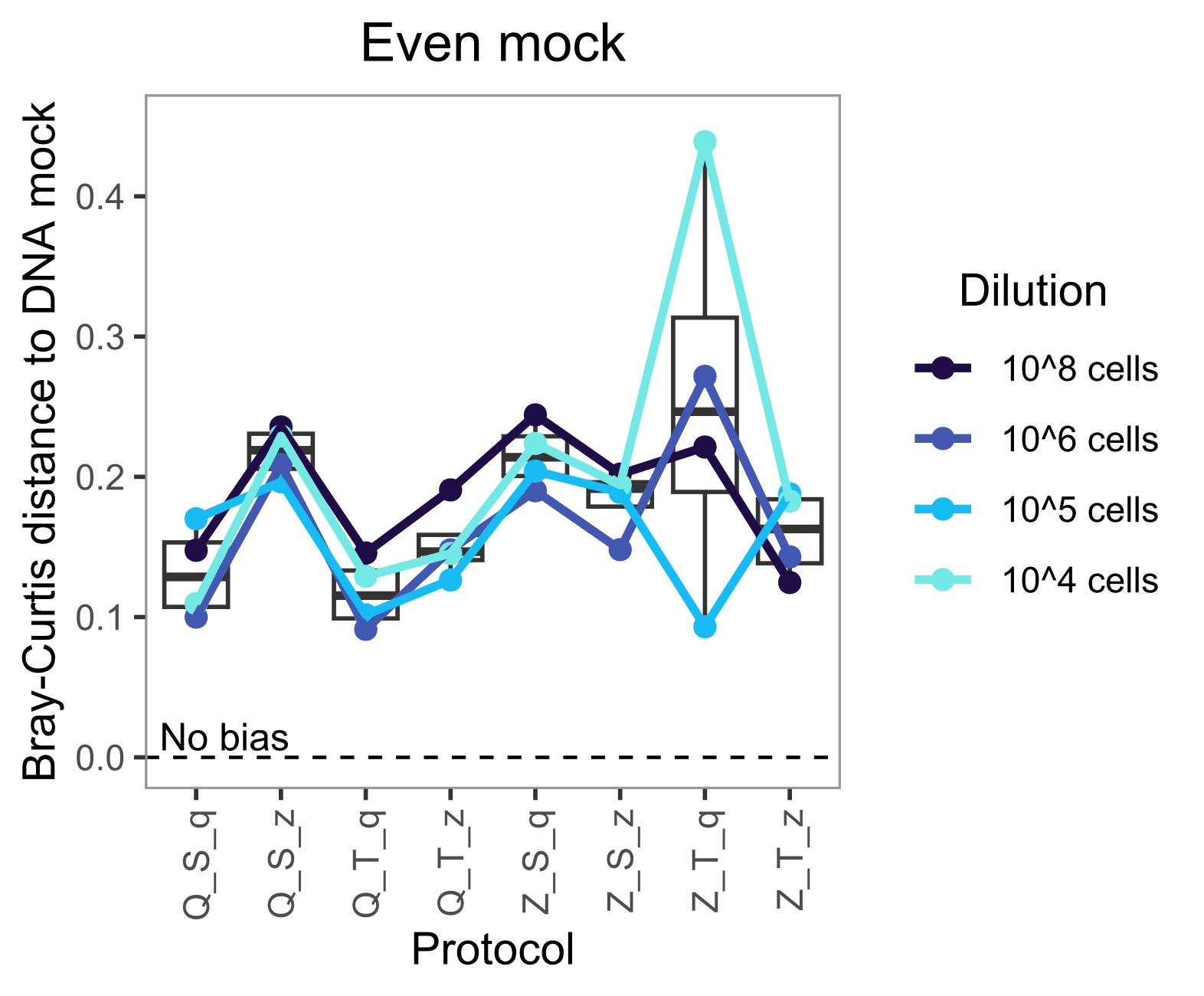
**

**C**

**A**

**B**

|  | **Even mock** | | | | | **Staggered mock** | | | | |
| --- | --- | --- | --- | --- | --- | --- | --- | --- | --- | --- |
|  | Bray-Curtis | | Aitchison | | **Median rank** | Bray-Curtis | | Aitchison | | **Median rank** |
| **Protocol** | Distance | Rank | Distance | Rank |  | Distance | Rank | Distance | Rank |  |
| **Q_S_q** | 0.13 | 2 | 1.01 | 2 | **2** | 0.11 | 5 | 0.81 | 5 | **5** |
| **Q_S_z** | 0.22 | 7 | 1.96 | 8 | **7.5** | 0.19 | 8 | 1.24 | 8 | **8** |
| **Q_T_q** | 0.12 | 1 | 0.91 | 1 | **1 (best)** | 0.09 | 3 | 0.80 | 3 | **3** |
| **Q_T_z** | 0.15 | 3 | 1.12 | 3 | **3** | 0.10 | 4 | 0.81 | 4 | **4** |
| **Z_S_q** | 0.21 | 6 | 1.61 | 5 | **5.5** | 0.17 | 7 | 1.13 | 7 | **7** |
| **Z_S_z** | 0.19 | 5 | 1.39 | 4 | **4.5** | 0.14 | 6 | 0.96 | 6 | **6** |
| **Z_T_q** | 0.25 | 8 | 1.78 | 6 | **7** | 0.05 | 2 | 0.52 | 2 | **2** |
| **Z_T_z** | 0.16 | 4 | 1.81 | 7 | **5.5** | 0.02 | 1 | 0.41 | 1 | **1 (best)** |

**Supplementary Figure S6. The magnitude of extraction bias varies between extraction protocols, but also between mock communities.** Extraction bias, measured as Bray-Curtis distance to the DNA mock composition, varies between extraction protocols in the even (**A**) and staggered (**B**) mock community, with no protocol achieving a perfect representation (no bias). Substantial differences in protocol bias were observed between the two mock communities, independent of the chosen distance measure (**C**). Boxes (**A**, **B**) denote the median and interquartile range (IQR), whiskers represent values up to 1.5 times the IQR, and dots indicate individual samples. A darker red background color (**C**) indicates higher extraction bias per protocol.

**Staggered mock, corrected by 10^6^ even sample**

**Even mock, 10^6^ sample corrected by itself**

**Even mock, other samples corrected by 10^6^ even sample**

**Spike-in mock, corrected by 10^6^ even sample**


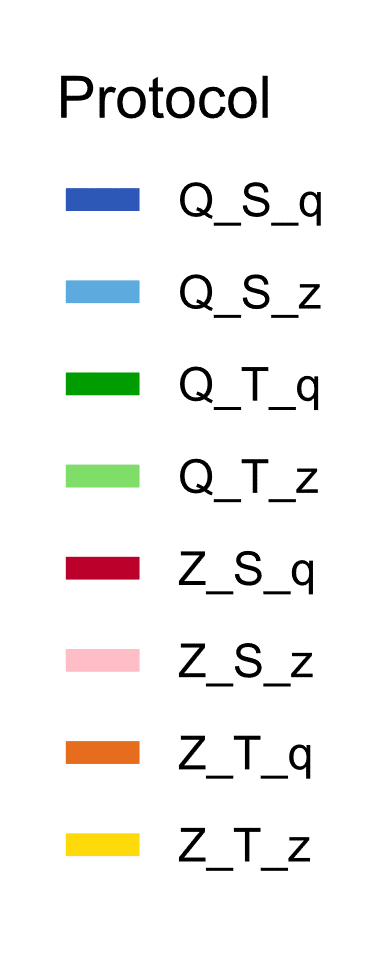

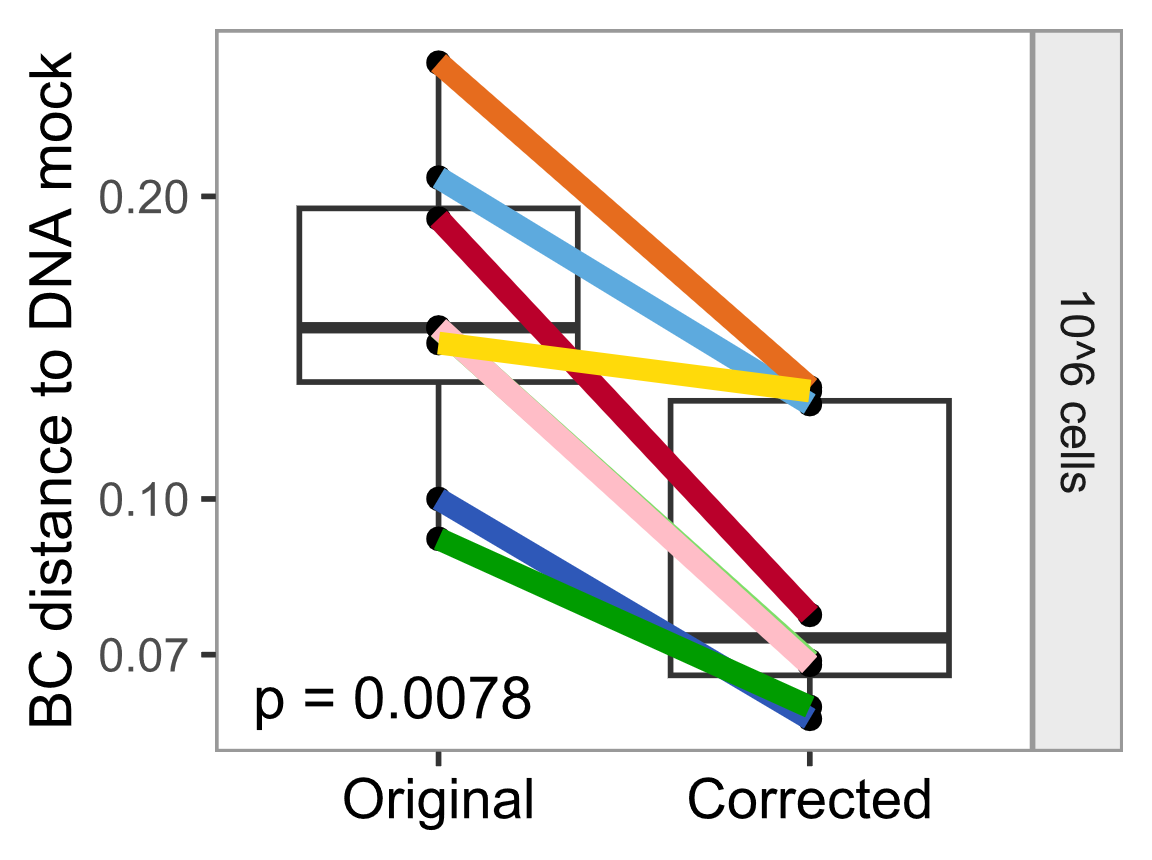

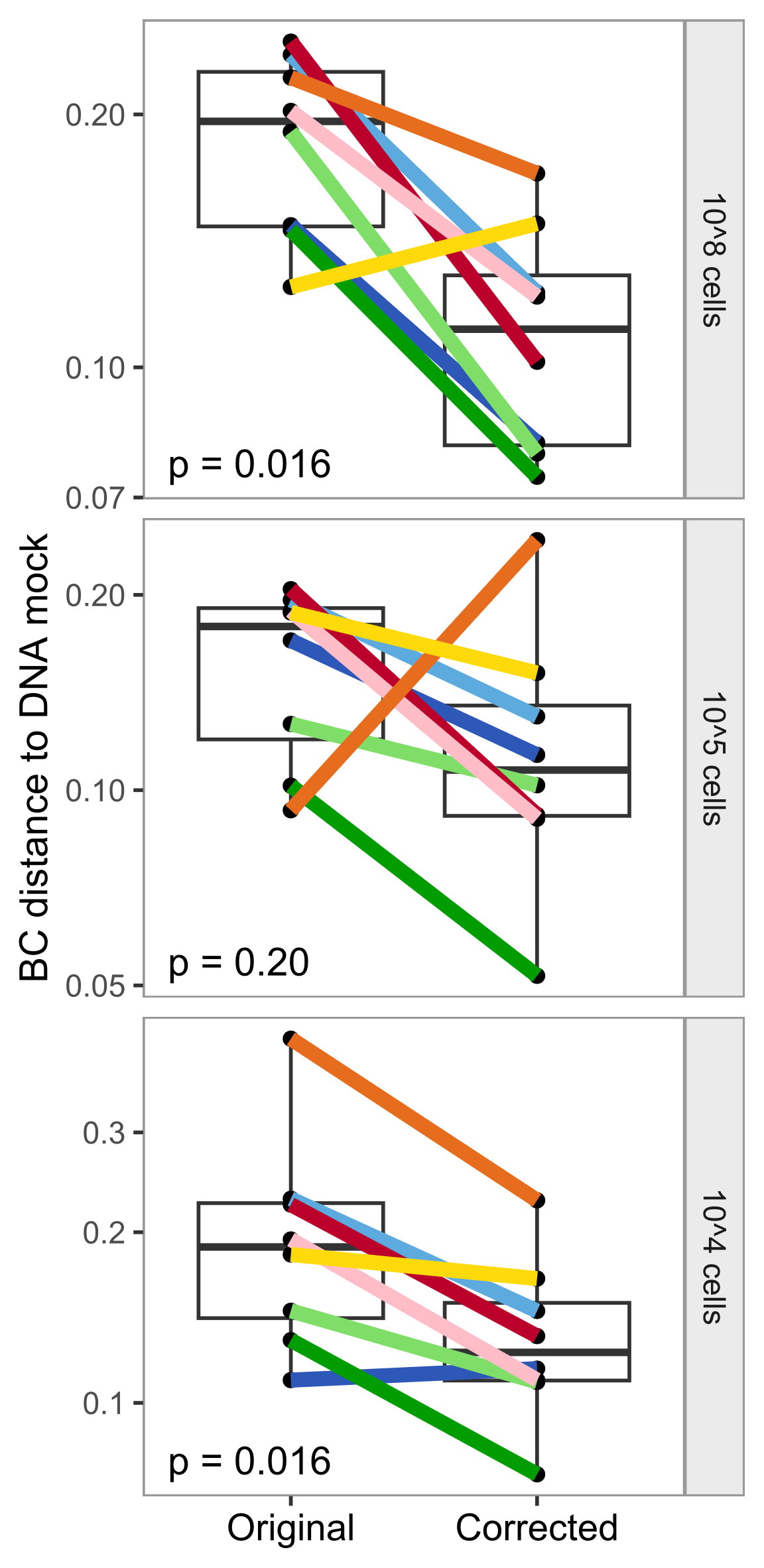

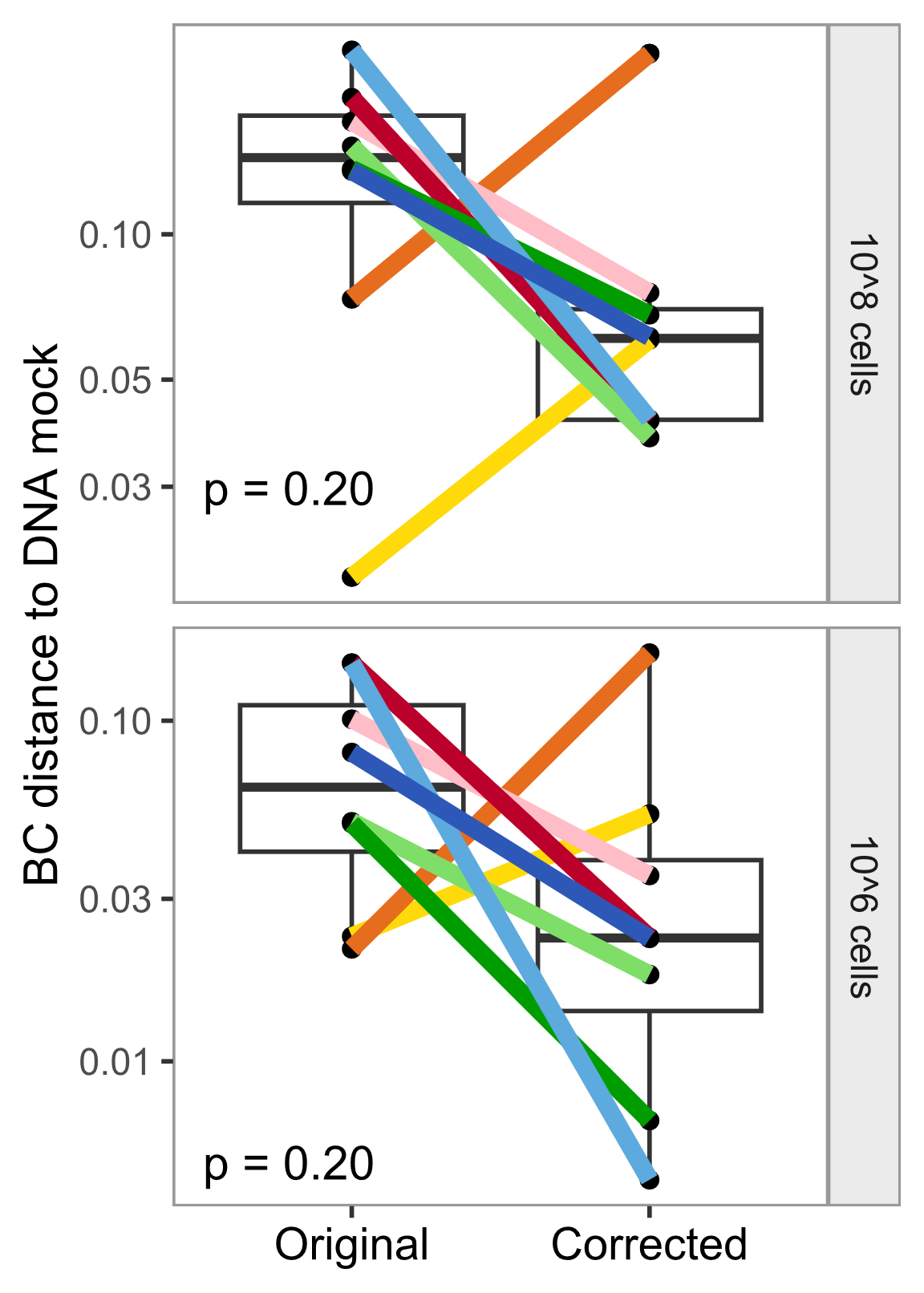

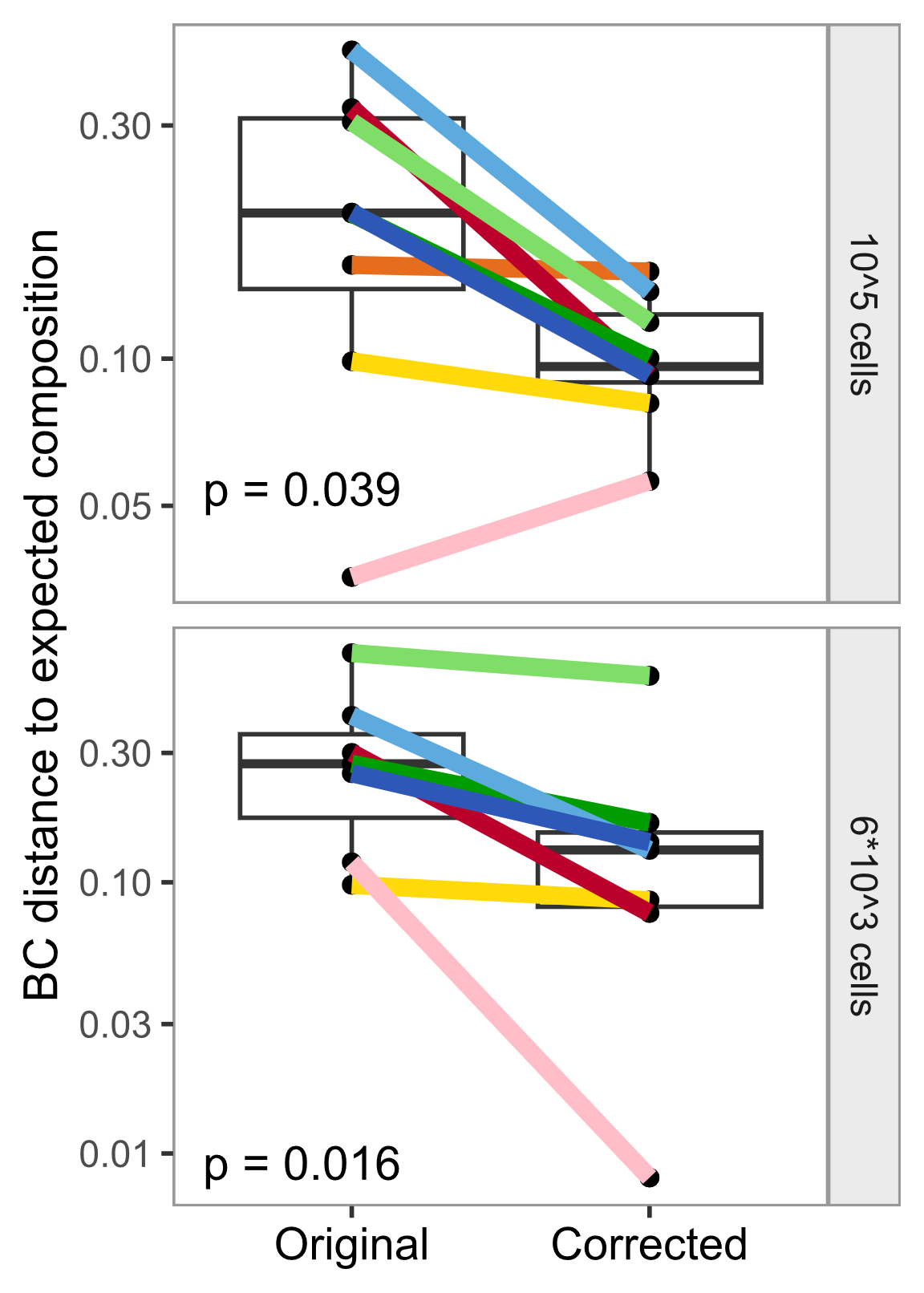


**A**

**B**

**C**

**D**

**Supplementary Figure S7. Distance to the DNA or expected mock composition is reduced after applying morphology-based correction of extraction bias in all mock communities, shown for all protocols.** Extraction bias per protocol was calculated from the 10^6^ even mock samples, summarized by bacterial morphology group, and applied to the 10^6^ even mock sample (internal correction, **A**), but also to different samples of the same mock (even mock 10^8^, 10^5^, 10^4^ cells, **B**), different samples of a different mock (staggered mock, **C**), and to different samples with different taxa (spike-in mock, **D**). Extraction bias was measured by Bray-Curtis dissimilarity to the DNA mock composition (even mock, staggered mock) or to the expected mock composition (spike-in mock). Boxes denote the median and interquartile range (IQR), whiskers represent values up to 1.5 times the IQR, dots indicate individual samples. Q: Qiagen extraction kit, Z: ZymoResearch extraction kit, S: ‘soft’ lysis condition, T: ‘tough’ lysis condition, q: Qiagen/Stratec buffer, z: ZymoResearch buffer.


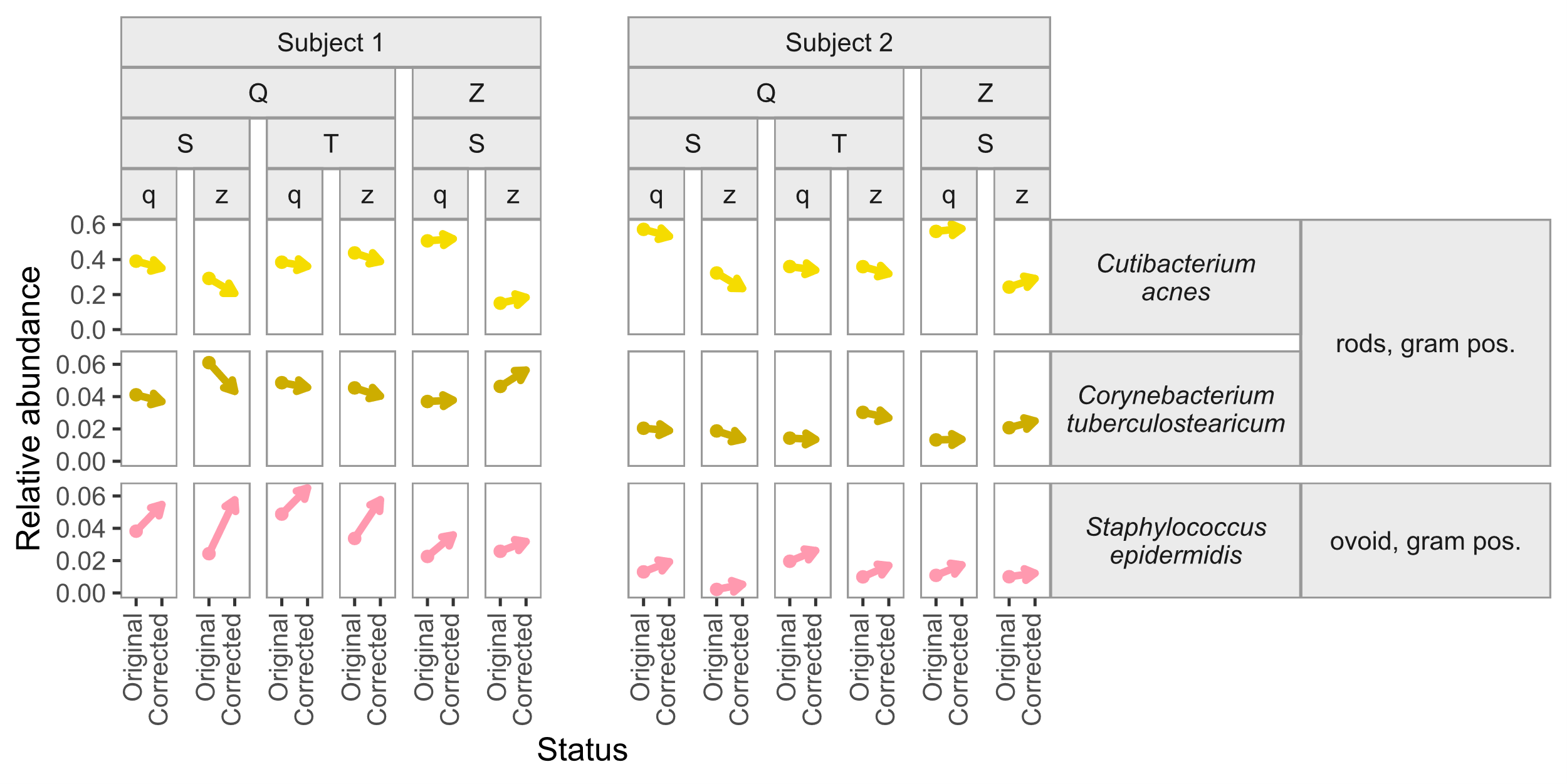

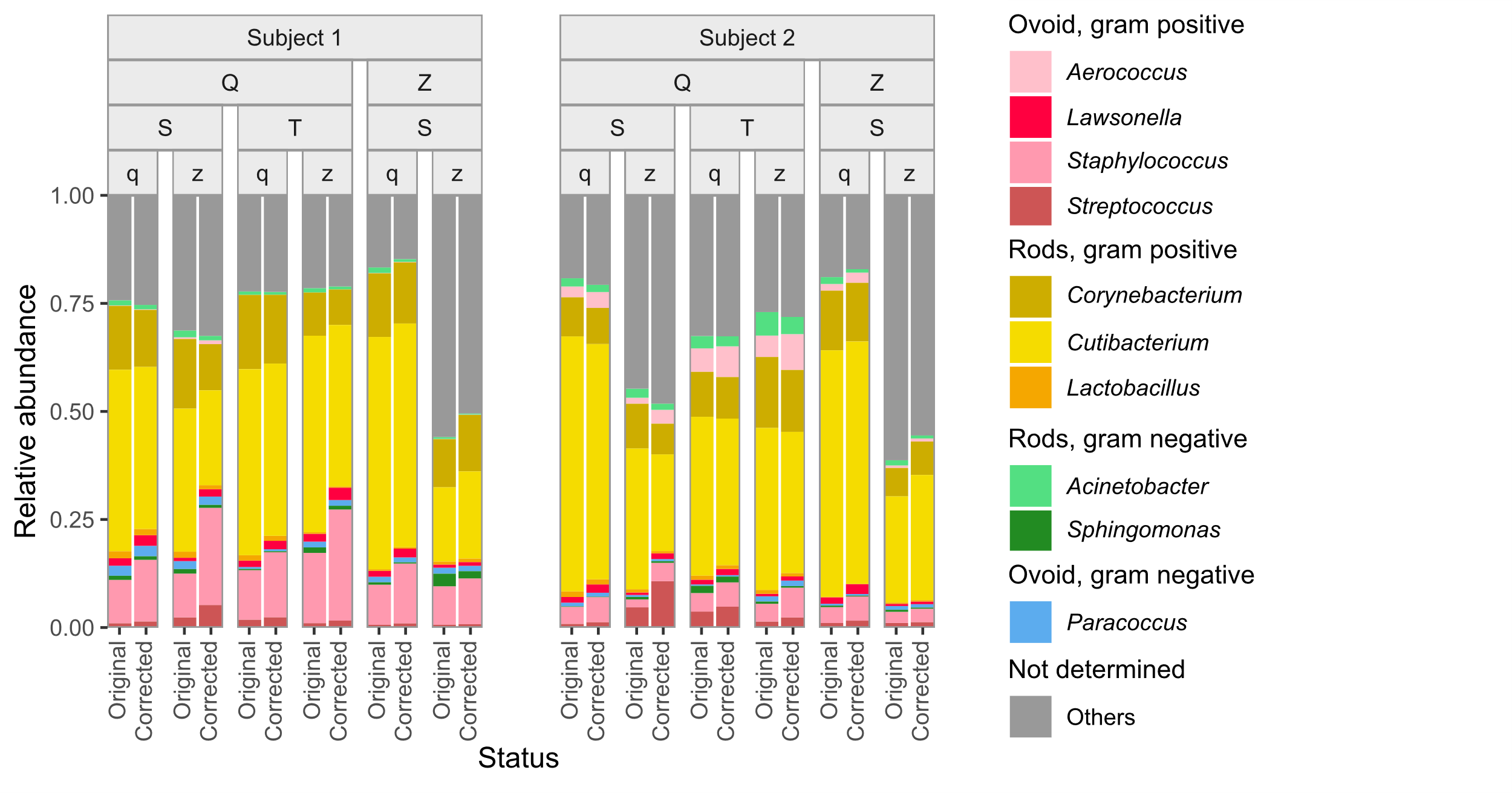


**A**

**B**

**Supplementary Figure S8. Impact of the bacterial morphology-based correction of extraction bias on skin microbiome samples**. Both the overall sample compositions on genus level (**A**) and the relative abundance of the top three skin microbiome taxa on species level (**B**) changed considerably after correcting for extraction bias. Sample composition (**A**) is shown for the top 10 skin genera, with the remaining genera summarized as Others. Q: Qiagen extraction kit, Z: ZymoResearch extraction kit, S: ‘soft’ lysis condition, T: ‘tough’ lysis condition, q: Qiagen/Stratec buffer, z: ZymoResearch buffer.

**Supplementary Table S1. Correction factors of extraction bias per protocol and taxon, based on bacterial morphology group**. Darker pink background color indicates more severely underrepresented taxa that are corrected up, and darker blue background color indicates more severely overrepresented taxa that are corrected down in their relative abundance by multiplying with the factor presented in the table. Q: Qiagen extraction kit, Z: ZymoResearch extraction kit, S: ‘soft’ lysis condition, T: ‘tough’ lysis condition, q: Qiagen/Stratec buffer, z: ZymoResearch buffer.

|  | **Bacterial morphology group** | | |
| --- | --- | --- | --- |
| **Protocol** | **ovoid, gram pos.** | **rods, gram pos.** | **rods, gram neg.** |
| **Q_S_q** | 1.36 | 0.86 | 0.82 |
| **Q_S_z** | 2.13 | 0.64 | 0.63 |
| **Q_T_q** | 1.31 | 0.92 | 0.81 |
| **Q_T_z** | 1.61 | 0.84 | 0.7 |
| **Z_S_q** | 1.69 | 1.09 | 0.56 |
| **Z_S_z** | 1.32 | 1.3 | 0.64 |
| **Z_T_q** | 0.53 | 1.93 | 1.21 |
| **Z_T_z** | 0.74 | 1.95 | 0.87 |
